# miR-30d-5p promotes beta cell recovery and immunomodulation in type 1 diabetes

**DOI:** 10.3389/fendo.2026.1838666

**Published:** 2026-06-02

**Authors:** Laia Gomez-Muñoz, David Perna-Barrull, Dagmar Klein, Silvia Alvarez-Cubela, Gerard Godoy-Tena, Daniel A Cook, Catalina Quimper Voto-Bernales, Mayur Doke, Marta Murillo, Aina Valls, Ricardo Luis Pastori, Juan Dominguez-Bendala, Marta Vives-Pi

**Affiliations:** 1 Immunology Department, Germans Trias i Pujol Research Institute (IGTP) and University Hospital (HGTiP), Autonomous University of Barcelona, Badalona, Spain; 2 Diabetes Research Institute, University of Miami Miller School of Medicine, Miami, FL, United States; 3 Laboratory of Tumor Inflammation and Angiogenesis, Center for Cancer Biology, VIB, Leuven, Belgium; 4 Laboratory of Tumor Inflammation and Angiogenesis, Center for Cancer Biology, Department of Oncology, KU Leuven, Leuven, Belgium; 5 Pediatrics Department, Germans Trias i Pujol Research Institute (IGTP) and University Hospital (HGTiP), Autonomous University of Barcelona, Badalona, Spain

**Keywords:** autoimmunity, beta-cell, epigenetic regulation, immunoregulation, miRNA, type 1 diabetes

## Abstract

**Background:**

Type 1 diabetes (T1D) is an autoimmune disease characterized by the destruction of insulin-producing beta cells, leading to hyperglycemia. A transient partial remission (PR) phase, marked by improved glycemic control and suppressed autoimmunity, often occurs shortly after diagnosis. We hypothesized that miR-30d-5p, which is highly upregulated during the PR phase in children, may contribute to immune regulation and beta cell recovery.

**Methods:**

We investigated the role of miR-30d-5p using human pancreatic slices (HPSs), non-obese diabetic (NOD) mice and human peripheral blood samples. HPSs were transfected with miR-30d-5p oligonucleotides to examine post-transcriptional regulation mechanisms. RNA sequencing was conducted in HPSs to identify differentially expressed genes. Functional assays included insulin secretion, and lineage tracing of beta cells. Additionally, non-obese diabetic (NOD) mice were treated with miR-30d-5p to evaluate effects on diabetes onset and incidence. T cell phenotyping and cytokine secretion were assessed in human T cells from patients with T1D.

**Results:**

HPS transfection was successfully achieved, providing a model to investigate the effects of the miRNA in insulin-expressing cells. Functional studies suggested that miR-30d-5p contributes to insulin secretion, and lineage tracing was consistent with the emergence of insulin-producing cells. RNA sequencing identified transcriptional changes associated with pathways related to beta cell function and cellular differentiation. In NOD mice, miR-30d-5p treatment was associated with delayed diabetes onset, suggesting potential immunomodulatory and beta cell-protective effects. In human T lymphocytes, miR-30d-5p was associated with increased expression of inhibitory molecules (PD-1, CTLA-4, CD200, TIM-3, LAG-3) and modulated interferon-gamma secretion.

**Conclusions:**

Our findings suggest that miR-30d-5p may participate in in immune cell regulation and processes associated with beta cell recovery in T1D. This study highlights the complex interaction between immune and epigenetic mechanisms during the PR phase and supports further investigation of miR-30d-5p as a potential therapeutic target for preserving beta cell function.

## Introduction

Type 1 diabetes is an autoimmune disease with complex heterogeneous pathogenesis. Following initiation of insulin therapy, the majority of pediatric patients experience a period of partial remission (PR), commonly referred to as the “honeymoon” phase. During this stage, pancreatic beta cells experience a transient functional recovery with improved endogenous insulin secretion, which reduces the need for exogenous insulin and maintains better glycemic control. The mechanisms behind this phase are unclear but may involve reduced beta cell stress due to corrected hyperglycemia, as well as improved viability, insulin sensitivity, regeneration, and immune regulation (1). It is therefore important to understand not only the immunological and metabolic fluctuations that precede disease diagnosis but also those occurring after it. This can provide valuable insights into pathways leading to beta cell protection and immune modulation, impacting patient stratification, intervention timing, and therapeutic precision.

Epigenetic changes, defined as stable and heritable changes in gene expression that do not involve alterations of the DNA sequence, can mediate the effects of external surrounding environments by regulating DNA accessibility or repressing gene expression. Among the different epigenetic mechanisms, microRNAs (miRNAs) are small non-coding RNA molecules of 18–25 nucleotides that regulate gene expression at the post-transcriptional level in a sequence-specific manner. Due to their ability to regulate a wide range of cellular developmental processes—such as cellular proliferation, metabolism, cell death, and intracellular communication—excess or deficiency of miRNAs has been associated with numerous diseases, including cancer, neurodegenerative, cardiovascular, and autoimmune diseases such as type 1 diabetes (2, 3). Thus, investigating the epigenetic dynamics at PR may shed light on the underlying mechanisms driving this phase and help clarify the interplay between immune regulation and metabolic control (4).

The onset of islet autoimmunity has been associated with several dysregulated miRNAs that directly affect T cell activation, differentiation, and function as well as beta cell apoptosis, insulin secretion and endoplasmic reticulum stress (5–7). This suggests a direct role of these molecules in regulating the development of immune-mediated islet cell autoreactivity. However, only a few studies have addressed changes in miRNA levels over time upon diagnosis (8, 9). Specifically, in children, one of the first studies found that miR-25 levels in sera were associated with improved glycemic control and stimulated C-peptide secretion three months after diagnosis (10). Similarly, levels of the miR-23~24~27 cluster in newly diagnosed children predict C-peptide loss over time and are upmodulated upon disease progression (11). Additionally, it was found that at three months—when PR usually occurs—the miR-197-3p was the strongest predictor of residual beta cell function one year after diagnosis in children with type 1 diabetes (12).

We previously reported that pediatric patients with type 1 diabetes exhibit a distinct plasma miRNA profile during the PR phase in comparison to non-remitter patients. Among the differentially expressed miRNAs, miR-30d-5p showed the strongest upregulation during remission and was linked to immune regulation, apoptosis, metabolism and regenerative pathway (13). We demonstrated that its inhibition in non-obese diabetic (NOD) mice can modulate various immunoregulatory parameters such as regulatory T cell (Treg) levels or PD-1 and CD200 expression, worsening the ongoing autoimmune attack. MiR-30d-5p is a glucose-regulated miRNA that has been associated with both the induction of insulin production and the protection of beta cell function from impairment caused by proinflammatory cytokines (14, 15). It is known to be highly expressed by pancreatic beta cells, suggesting that they are likely one of its primary sources (14). Metabolically, this miRNA influences insulin signaling, adipocyte differentiation, and glucose metabolism, and it is implicated in type 2 diabetes and gestational diabetes (16). Moreover, it has been associated with protection from inflammation (17) and autoimmunity (14) since it can regulate cytokine production, macrophage polarization, and activation of inflammatory pathways such as NF-κB.

In this study, we use human blood and pancreatic tissue to investigate miR-30d-5p’s role in T-cell regulation and insulin-expressing cells. We show that miR-30d-5p is involved in the regulation of inhibitory molecule expression in T lymphocytes and interferon-gamma (IFN-γ) secretion. Lineage tracing and dynamic perifusion of human pancreatic slices (HPSs) suggest that miR-30d-5p is associated with the emergence of insulin-expressing cells and contributes to changes in insulin synthesis and secretion through gene modulation. Importantly, miR-30d-5p contributes to delay autoimmune diabetes onset in NOD mice by sustaining immune modulation throughout follow-up. These findings highlight miR-30d-5p as a potential biomarker and therapeutic target for monitoring and modulating immune responses and beta cell function in type 1 diabetes.

## Materials and methods

### Human pancreatic tissue slicing and culture

HPSs were obtained or cut from non-diabetic deceased donors’ pancreatic tissue following the protocol in (18). After slicing, they were cultured up to 12 hours in human slice culture medium on AirHive dishes (custom-made by Biorep, Miami, FL) at 30 °C with 5% CO2. The medium contains BrainPhys neuronal basal medium (Stemcell Technologies, Vancouver, BC) supplemented with 2% B27 minus insulin, 1% penicillin-streptomycin-amphotericin B, 1% Glutamax (Invitrogen), 3.7 mg/mL L-Glutamic Acid (Sigma Aldrich), 5.5 mM D-glucose (BrainPhys already has 2.5 mM), 100 mg/mL trypsin inhibitor (Glycine max), 10 mg/mL aprotinin, 10 mg/mL chymostatin, and 1% HEPES buffer (Invitrogen). Cell viability was assessed by live/dead staining (Live/Dead Cell Imaging Kit [488/570], Thermo Fisher) and mitochondrial staining (BioTracker™ 633 Red Mitochondria Dye, Sigma-Aldrich), with imaging on a fluorescent microscope (ApoTome Axiovert 200M, Zeiss).

### Adenovirus transduction and BMP-7 treatment

Recombinant adenoviruses were constructed using the serotype 5 adenovirus with an E1/E3 deletion. To track insulin-expressing cells, slices were co-transduced after 24 h with Ad-(CMV)- loxP-dsRED-loxP-BFP-2A-GCaMP6 and Ad-HIP-Cre (Vector Biolab, Malvern, PA, USA), as described (18). Cre expression driven by the human insulin promoter in the tracer virus induces recombination in the reporter virus, excising dsRed and tagging cells blue (BFP). When needed, human bone morphogenetic protein 7 (BMP-7) (PeproTech, 100 pg/mL) was added as a positive control for regeneration. Medium was removed, 2 mL of virus (with or without BMP-7) was added per dish, and slices were incubated for 24 h at 30 °C. The next day, medium was replaced, and BMP-7 treatment continued for four more days (5 days in total).

### Transfection with miR-30d-5p inhibitor or mimic and efficiency determination

Two days post-transduction (or one day post-slicing for RNA-seq/perifusion), HPSs were transfected with miRCURY LNA miRNA mimics/inhibitors or controls. Inhibitor (300 nM) or mimic (200 nM) probes (Exiqon) were prepared in BrainPhys medium (no serum/antibiotics). DharmaFECT 2 was diluted (10-fold for mimics, 6.5-fold for inhibitors) in parallel, then combined with the probes after 5 min and incubated for 30 min at RT. The 400 μL complex was added to 1,600 μL of antibiotic-free slice culture medium ± BMP-7. To assess transfection efficiency, slices were transfected with 6-FAM-labeled miR-30d-5p inhibitor and stained. After 24 h, slices were fixed (4% paraformaldehyde, PFA), permeabilized (0.3% Triton), and blocked. Insulin was stained with a guinea pig anti-insulin antibody (48 h, 4°C), followed by Alexa Fluor 647 anti-guinea pig and DAPI (24 h, 4°C). Slices were imaged on a Leica SP5 confocal microscope and reconstructed in Z-stacks (15–30 slices, 2.5–5.0 μm). Using ImageJ/FIJI, insulin^+^ and DAPI^+^ cells were quantified to evaluate 6-FAM^+^ transfection. Moreover, the expression of miR-30d-5p after the transfection of its inhibitor or mimic was assessed by RT-qPCR. Relative values were calculated with the 2^−ΔΔCt^ method, and gene expression was normalized to the housekeeping gene *U6 snRNA* (Assay ID: 001973).

### Tracking insulin-producing cell emergence

After 5 days of BMP-7 treatment, slices were kept in complete medium for another 5 days. The slices were kept in complete culture medium for another 5 days. Throughout this period, daily imaging was conducted at 10X magnification, focusing on specific regions of interest (ROIs). These ROIs were selected on the initial treatment day, using distinct topographical landmarks within the slice to ensure consistent and accurate imaging over time. Images were captured in bright field, red and blue channels using a fluorescent microscope (ApoTome Axiovert 200M, Zeiss). Images of randomly selected islets were analysed by using ImageJ/FIJI. The blue (BFP) signal, indicative of pre-existing β-cells in the HPSs, is generally detected between 72 and 96 hours, with these cells showing no red fluorescence at any stage. The dsRED^+^ cells are non-insulin-producing cells (red). Emerging insulin-producing cells, which are primarily observed after BMP-7 removal, are dsRED cells that also begin to express BFP, suggesting a late recombination event associated with the onset of new insulin expression. Then, the transient violet (red+blue) color of these cells can be used to identify emerging insulin-producing cells.

### Bulk RNA library preparation, sequencing and analysis

RNA was extracted from HPSs (miRNeasy, Qiagen) and processed with the NuGEN Universal Plus mRNA-Seq kit (100 ng input, 15 PCR cycles). Libraries were quality-checked (Qubit, BioAnalyzer) and sequenced (150 bp paired-end, >50M reads/sample) on the Illumina NovaSeq X. Reads were pseudo-aligned to the human reference genome (hg38) using kallisto (19) and transcript abundances were summarized to the gene level using tximport. Differential expression was analyzed with DESeq2 (20), including donor as a covariate (design = ~ Donor + Condition). Variance-stabilized values were corrected for donor effects with ComBat (sva), then principal component analysis (PCA) and hierarchical clustering were performed. Inhibitor and mimic controls were combined into a single group for differential expression testing. Differentially expressed genes (DEGs) were defined as protein-coding genes with |log_2_ fold change| >0.5 and FDR <0.05. Gene ontology (GO) enrichment was done with Enrichr (https://maayanlab.cloud/Enrichr/, accessed 23 Aug 2024), with p ≤ 0.05 considered significant. Transcription factor activity was inferred using DoRothEA (21), limited to regulons with confidence A–C, with q ≤ 0.05 and |NES| ≥ 2 as significant. Data visualization and statistical plots were generated in R (ggplot2, ggpubr), with analyses performed in R v4.3.2.

### Dynamic glucose-stimulated insulin release

Dynamic perifusion is the gold standard for assessing real-time, glucose-regulated insulin release *in vitro* (22). Pancreatic slices from nPOD (n=3) were transfected with 300 nM miR30d-5p inhibitor or control inhibitor. Transfection reagent (Qiagen) remained in culture for 48 hours. Slices were cultured 8 days at 30°C, including an untreated control. On perifusion day, slices were incubated 90 min at 37°C in Krebs buffer with 3 mM glucose, then loaded into single-slice chambers (23). Six viable slices per condition were perfused in closed chambers (Warner Instruments) using an automated system (Biorep PERI4) at 100 µL/min, collecting fractions every 2 min. After a 60 min wash (3 mM glucose), slices were sequentially exposed to 3 mM (10 min), 16.7 mM (20 min), 1 mM glucose (30 min), 30 mM KCl (5 min), and 3 mM glucose (10 min). Insulin was measured by ELISA (Mercodia Inc., cat #10-1113-01), normalized to KCl-stimulated release.

### Monitoring autoimmune diabetes onset in NOD mice

Wild-type NOD mice were obtained from Jackson Laboratory (Bar Harbor, ME, USA) and maintained in our facility under specific pathogen-free conditions, with a 12 h light/dark cycle, controlled temperature and humidity, and ad libitum access to water and irradiated Teklad Global 18% Protein Rodent Diet (Harlan, Indianapolis, IN, USA). Only prediabetic 8-week-old NOD females were used. Glycosuria was monitored twice weekly with urine test strips (Combiscreen, Analyticon Biotechnologies AG, Lichtenfels, Germany) until 23 weeks, and autoimmune diabetes was confirmed when blood glucose exceeded 300 mg/dL (OneTouch Verio Reflect^®^, LifeScan IP Holdings, LLC., Zug, Switzerland).

### *In vivo* miR-30d-5p administration and autoimmune diabetes incidence

The mature mmu-miR-30d-5p sequence (5’-UGUAAACAUCCCCGACUGGAA-3’) was obtained from www.mirbase.org and is conserved in mice and humans. Mice were treated with *in vivo* miR-30d-5p mimic or control (5’-UCACAACCUCCUAGAAAGAUAG-3’, miRIDIAN, Dharmacon) using *in vivo*-jetPEI (#101000030) for transfection. Three groups of ≥9 normoglycemic 8-week-old NOD mice received 1) miR-30d-5p, 2) control miRNA, or 3) vehicle (jetPEI). Mice were given three i.p. injections every three days of 200 μL 5% glucose containing 80 μg miRNA (0.4 μg/μL) and jetPEI at N/P = 8. At study end or upon diabetes onset, spleens were harvested and processed.

### Spleen immunophenotyping

To determine changes in the percentage of different immune cell subpopulations after miR-30d-5pupregulation, the spleen of mice was immunophenotyped by flow cytometry. Splenocytes were obtained by mechanical disruption (13) counted and stained using two panels (**Supplementary Table 1**). For the Leukocyte Panel, cells were incubated 20 min with antibodies, washed with PBS, and stained with 4 μL of 7-aminoactinomicina D (7-AAD, BD Biosciences) in 100 μL PBS for 10 min before acquisition. For the T Lymphocyte Panel, cells were surface-stained with antibodies and 10 μL Brilliant Stain Buffer (BD Biosciences) for 20 min, then intracellularly stained with FOXP3 FITC using the True-Nuclear™ Transcription Factor Buffer Set (BioLegend) for 40 min at 4°C protected from light. Samples were washed twice, and ≥200,000 leukocyte events per sample were acquired on FACS LSR Fortessa (BD Biosciences). Necrotic/apoptotic cells were excluded by FSC-A/SSC-A and 7-AAD, doublets by FSC-A/FSC-H, and FMO controls were used. Data were analyzed with FlowJo (Tree Star Inc., Ashland, OR, USA).

### Human samples

For the identification of the effect of miR-30d-5p on primary human T lymphocytes, venous peripheral blood was obtained from 7 recent-onset type 1 diabetes pediatric patients and 6 age- and sex-matched non-diabetic controls at the Germans Trias i Pujol University Hospital. To evaluate miR-30d-5p expression in T-cell subsets, additional samples were collected from 10 patients during the PR phase and 6 non-remitters (**Supplementary Table 2**). Blood (6–10 mL) was collected in EDTA tubes and processed within 6 h. All patients met the American Diabetes Association classification criteria for type 1 diabetes (24), with at least one positive anti-islet autoantibody at onset. Inclusion criteria were age 4–18 years and a normal body mass index according to Spanish pediatric charts (25). Exclusion criteria were being under immunosuppressive or anti-inflammatory treatment, type 2 diabetes, pregnancy, compromised kidney function, or liver diseases. PR was defined using IDAA1c: HbA1c (%) + [4 × insulin dose (U/kg/day)], with values ≤9 indicating PR. Patients not meeting PR criteria after 8 months were classified as non-remitters.

To dissect the effect of the PR phase-upregulated miR-30d-5p on cell function and processes consistent with the emergence of insulin-expressing cells, human pancreatic tissue biopsies from eight nondiabetic deceased donors (five male and three female; age range 2–55 years) were obtained from the cGMP facility at the Diabetes Research Institute (DRI), University of Miami. HPSs were also obtained from the nPOD program (University of Florida) or Prodo Labs (Aliso Viejo, CA).

### Clinical and laboratory testing

Clinical descriptors on each patient and control subject were collected, including age, sex, and body mass index. Blood samples from patients with type 1 diabetes were obtained for centralized measurement of HbA1c and basal non-fasting C-peptide, which reflects residual insulin storage. HbA1c was determined by high-performance liquid chromatography (ADAMS A1c HA-8180V, Arkray, MN, USA) and basal non-fasting C-peptide was determined by ELISA (Architect i2000, Abbott, IL, USA). Insulin requirements were also recorded.

### PBMC isolation, freezing and viability

PBMCs were obtained through Ficoll Paque (GE Healthcare Life Sciences, Marlborough, MA, USA) density gradient centrifugation. The obtained PBMCs were frozen at 10-20×10^6^ cells/mL in a cryopreservation medium prepared with cold FBS + 10% dimethyl sulfoxide (DMSO, Sigma-Aldrich, St. Louis, Missouri, USA) for later use. To assess cell viability and counting, 10 μL of cells were incubated with 2 μL of 7-AAD in 50 μL of PBS for 10 min at room temperature and protected from light. Then, 10 μL of Perfect Count Microspheres (Cytognos SL) were added to perform cell counting. Cells were acquired by FACSCanto II flow cytometer (BD Biosciences) using the FACSDiva software (BD Biosciences).

### T-cell subsets sorting

To determine if lymphocytes express miR-30d-5p and which T-cell subset (cytotoxic CD8, conventional CD4, or regulatory) has higher levels, fresh PBMCs were used for sorting. PBMCs from 10 mL blood were washed and resuspended in 200 μL FACSflow with antibodies: 7.5 μL CD3 V450, 3.75 μL CD4 PerCPCy5.5, 7.5 μL CD8 APCH7, 15 μL CD25 PE, and 30 μL CD127 AF647. Cells were incubated 30 min at 4°C, washed twice with 3 mL FACSflow, then resuspended in 1 mL FACSflow for sorting using BD FACSAria™ II. CD4^+^ T cells, CD8^+^ T cells, and CD25^+^CD127^low^ Tregs were collected and cryopreserved as dry pellets. Subset purity exceeded 90% in all samples.

### Quantitative RT-PCR

For miR-30d-5p expression analysis, total RNA including miRNA was extracted using the miRNeasy Tissue/Cells Advanced Mini Kit (Qiagen). RNA quality and quantity were checked with the TapeStation 2200 (Agilent), then reverse transcribed using the TaqMan Advanced miRNA cDNA Synthesis Kit and Veriti^®^ Thermal Cycler (ThermoFisher Scientific). TaqMan Advanced miRNA assays for hsa-miR-30d-5p (478606_mir) and 5S rRNA (Hs03682751_Gh) were run in triplicate 15 μL PCRs with TaqMan™ Fast Advanced Master Mix. Relative expression was calculated by the 2^−ΔΔCt^ method, normalized to 5S rRNA. For *IRS-1* expression, RT-qPCR used TaqMan^®^ assays for *IRS-1* (Hs00178563_m1) and *ACTB*(Hs01060665_g1), with relative expression calculated by 2^−ΔCt^ and normalized to *ACTB*.

### T cell isolation and culture

PBMCs were thawed, and viability and cell counts were measured as above. T cells were isolated by negative magnetic selection using the Human Pan T Cell Isolation Kit (Miltenyi Biotech, Bergisch Gladbach, Germany) using manufacturer’s instructions. Cell counts and viability were determined with antibodies: 1.5 μL CD3 PE (UCHT-1), 0.5 μL CD4 APC (EDU-2), 0.5 μL CD8 FITC (HIT8a) (Immunotools). CD3 purity exceeded 70% in all samples. T cells were cultured in 24- or 6-well flat-bottom plates at 0.5–1×10^6 cells/mL in complete RPMI-1640 medium supplemented with 2 mM L-Glutamine, 1 mM sodium pyruvate, 5% male human AB serum (Biowest), 100 IU/mL penicillin, 100 μg/mL streptomycin, and 0.025 mM 2-Mercaptoethanol. Cytokines IL-2 (100 U/mL), IL-7 (5 ng/mL), and IL-15 (5 ng/mL) were added (PreproTech, Cranbury, NJ, USA). T cells were stimulated with Dynabeads™ Human T-Activator CD3/CD28 beads (ThermoFisher Scientific) at a 1:1 bead-to-cell ratio, then incubated at 37°C with 5% CO_2_ for three days before electroporation.

### T lymphocyte electroporation

T cell suspension was placed in a sterile flow cytometry tube inside an EasySep Magnet (STEMCELL Technologies) for 2 minutes to separate beads from cells. Cells were washed at 400×g for 5 minutes at room temperature, and the supernatant containing medium with antibiotics was fully removed to prevent antibiotic toxicity during electroporation. Cells were resuspended in PBS + 2% FBS, and viability and counts were checked. The required volume of cells was then taken to electroporate at 5–10×10^6 cells/mL, washed, and resuspended in 150–200 μL Gene Pulser Electroporation Buffer (Bio-Rad, Hercules, CA) containing 500 nM miRNA inhibitor (hsa-miR-30d-5p) or negative control probe A (Exiqon, Copenhagen, Denmark), depending on the condition. Electroporation was performed using the Gene Pulser Xcell (220 V, 2 ms, 1 pulse, Bio-Rad) with 0.2 cm gap cuvettes (Bio-Rad). After electroporation, cells were transferred to pre-warmed recovery medium without antibiotics [RPMI-1640 (Biowest) supplemented with 2 mM L-Glutamine (Sigma), 1 mM sodium pyruvate (ThermoFisher Scientific), 10% male human AB serum (Biowest), and 0.025 mM 2-Mercaptoethanol (Sigma)], rested for ≥4 hours at 37°C in 5% CO_2_, washed, and cultured in 24-well flat-bottom plates with complete medium plus 100 U/mL IL-2 (PeproTech) for one day.

### Transfection efficiency

T cells were transfected as above using a 6-FAM–labeled miR-30d-5p inhibitor (Exiqon). To assess transfection efficiency, cells were collected at 4 and 24 h, stained with 1 μL CD3-PE (Immunotools) and 4 μL 7-AAD (BD Biosciences) in 100 μL PBS at 4°C for 15 min, and the percentage of 6-FAM positive cells was analyzed on a 3-laser FACS Canto II (BD Biosciences). Mock-electroporated cells (no oligonucleotide) served as a negative control. For miR-30d-5p expression analysis, we followed the same protocol as detailed in *Quantitative RT-PCR* section.

### T cell phenotype

At 24 hours post-culture, T cell activation, maturation, and immune checkpoint expression were assessed. Cell viability and counts were measured as before, using 0.2×10^6 cells per panel. Cells were first stained with Fixable Viability Stain 575V (1:1000, BD) in PBS for 15 minutes at room temperature in the dark, then washed and surface-stained with antibody mixes (100 μL, 20 minutes, 4°C). Panel 1 included CD3 V500, CD4 V450, CD8 APC-H7, CD25 PE, CD69 BV711, CCR7 PECy7, and CD45RA BV605 for surface staining, with intracellular FoxP3 FITC staining. Panel 2 surface staining used CD3 V500, CD4 APC, CD8 APC-H7, CD200 PE, TIGIT BV605, TIM-3 PECy7, LAG-3 PECy5, PD-1 BV711, and CD226 FITC, with intracellular CTLA-4 BV421. Details on antibodies are in **Supplementary Table 3**. Intracellular staining was done using the True-Nuclear™ Transcription Factor Buffer Set (BioLegend). Fixed and permeabilized cells were incubated with 1.25 μL Human TruStain FcX Fc Receptor Blocking Solution (BioLegend) for 15 minutes at 4°C in the dark, then stained with monoclonal antibodies (FOXP3 FITC or CTLA-4 BV421) for 40 minutes at 4°C protected from light. Samples were washed twice, and at least 100,000 CD3^+^ events per sample were acquired on an LSR Fortessa flow cytometer (BD Biosciences). Doublets were excluded by FSC-A/FSC-H gating, and FMO controls were used for key markers. Data were analyzed with FlowJo (Tree Star Inc., Ashland, OR, USA).

### T cell proliferation

T lymphocytes were stained with CellTrace Violet (CTV, ThermoFisher) to monitor proliferation and cultured in triplicate in 96-well flat-bottom plates (2.5×10^5^ cells/mL, 200 μL) under three conditions: complete medium only (negative control), with 100 U/mL IL-2 (basal stimulation), or with 12.5 μL/mL ImmunoCult™ CD3/CD28/CD2 activator (STEMCELL) as a second TCR stimulus to further expand them. After 2 days at 37°C and 5% CO_2_, proliferation was assessed in CD4 and CD8 subsets. Cells were stained with 7-AAD (BD Biosciences), CD3 PE (clone UCHT1, Immunotools), CD4 APC (clone EDU-2, Immunotools), and CD8 FITC (clone HIT8a, Immunotools) in 25 μL, incubated 20 min at 4°C, washed, resuspended in 150 μL PBS, acquired on an LSR Fortessa and analyzed with FlowJo.

### T cell cytokine profile

Supernatants from T cells re-stimulated with ImmunoCult™ (STEMCELL) under mock, control inhibitor, or miR-30d-5p inhibitor conditions were collected. Cytokines (IFN-γ, IL-1β, IL-10, IL-17A, IL-21, IL-4, IL-6, IL-8, CCL5, TNF-α) were quantified using a Luminex^®^ multiplex kit, and TGF-β1 with a simplex kit after acid/neutralization pre-treatment to detect the active form (ThermoFisher). Samples were analyzed on a Luminex^®^ 200 system.

## Statistics

Data are presented as mean ± SD or SEM or as percentages, where appropriate. The distribution of continuous variables was tested for normality by the Kolmogorov-Smirnov test. For not-normally distributed variables, comparisons between two groups were performed by a non-parametric Mann-Whitney test and between three or more groups by Kruskal-Wallis with Dunn’s *post-hoc* test and for samples from the same donors subjected to different treatments, they were analyzed by repeated measures two-way ANOVA with the Geisser-Greenhouse correction and Tukey’s multiple comparisons test. Multivariate statistical analysis was performed using PCA. For autoimmune diabetes incidence in mice, a Mantel-Cox log-rank test was performed. For all tests, a two-tailed *p*-value of ≤0.05 was considered statistically significant. Levels of significance are indicated as: *, p ≤0.05; **, p ≤0.01; ***, p ≤0.001. Analyses were performed using the programs GraphPad Prism 10 (GraphPad Software Inc, San Diego, CA, USA) and R v4.1.0.

## Ethics

### Human samples

Experiments followed the Declaration of Helsinki and were approved by the Ethics Committee of Germans Trias i Pujol University Hospital (PI-19-010, PI-23-128), with written informed consent from participants or legal representatives. HPS procedures adhered to nPOD/OPPC SOPs and were approved by the University of Florida IRB (IRB201600029) and UNOS, with consent from each donor’s legal representative. Personal and clinical data were limited to what was necessary, handled confidentially, and complied with data protection laws.

### Mice

Animal experiments followed the Guide for the Care and Use of Laboratory Animals (Generalitat de Catalunya) and were approved by the Animal Experimentation Ethics Committee of CMCiB and IGTP and the Generalitat de Catalunya. Protocols adhered to 3R principles for animal welfare and were approved by the Ethics Committee of Germans Trias i Pujol Research Institute and Catalan Government (protocol 12723, 10 April 2025).

## Results

### Efficient delivery of miR-30d-5p inhibitor to human pancreatic slices suppresses target miRNA expression and is associated with altered beta cell function

**Figure 1:**
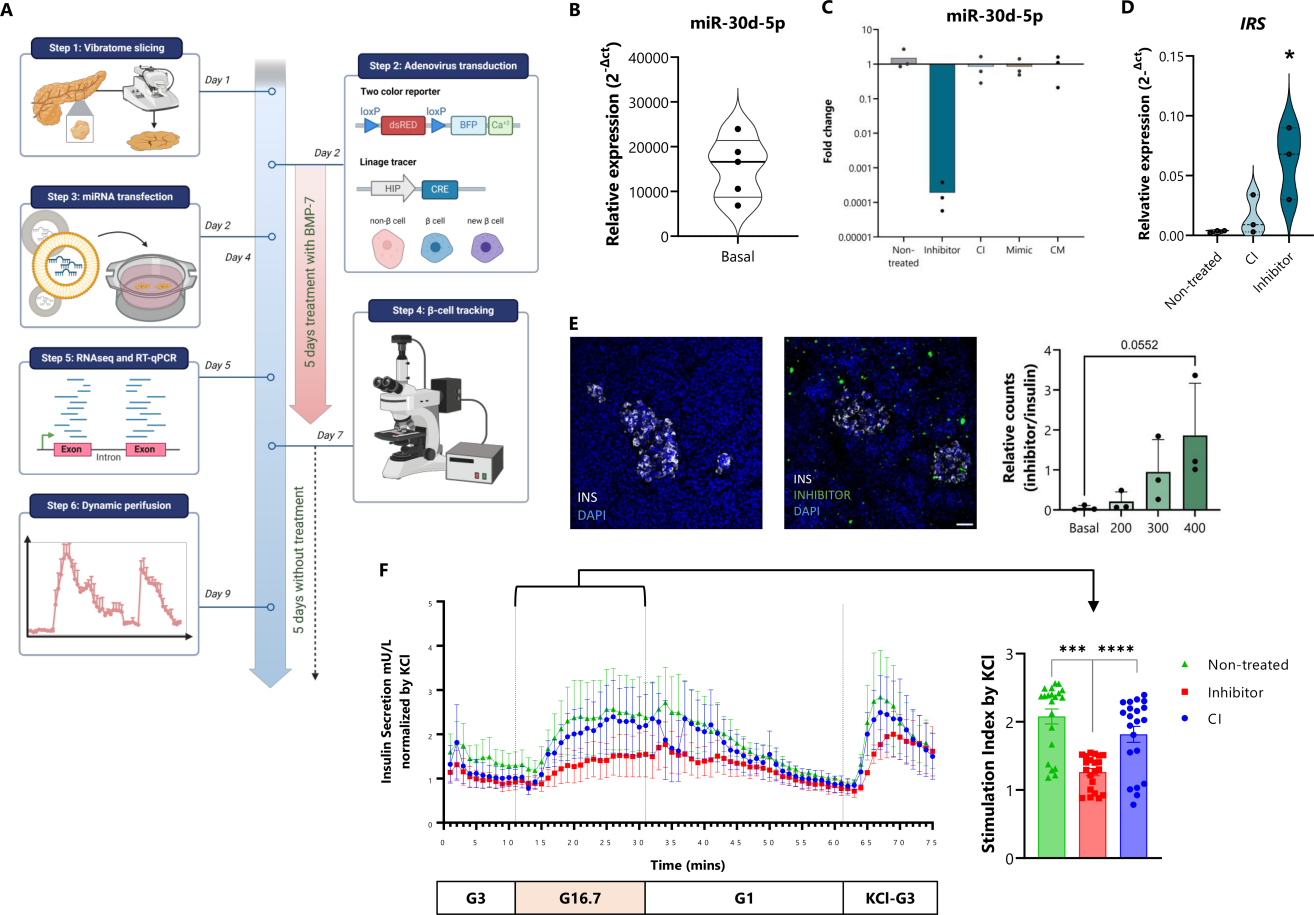
miR-30d-5p inhibitor’s delivery to human pancreatic slices efficiently blocks its target miRNA and impairs beta cell function. **(A)** Schematic overview of the protocol used for miRNA transfection in HPSs. **(B)** Relative expression levels of miR-30d-5p in HPSs just right after slicing and without treatment. MiR-30d-5p expression was normalized to U6 expression. Values are expressed as 2^−ΔCt^. Violin plot shows the median (thick line) and first and third quartiles (thin lines). N = 5 different pancreases. **(C)** Fold change in the miR-30d-5p expression using basal transcription (complete medium-cultured HPSs) as the standard value. Expression of miR-30d-5p was analyzed by single-assay RT-qPCR 72 hours after non-treatment or after transfection with 300 nM of miRNA inhibitor and control inhibitor (CI) or 200 nM of the miRNA mimic and control mimic (CM). The miRNA expression signal was normalized to U6 expression. Values are expressed as 2^-ΔΔCt^. Friedman test. N = 3 pancreases. **(D)** Relative expression of *IRS-1* following miR-30d-5p inhibition. Gene expression signal was normalized to *ACTB*. Values are expressed as 2^-ΔCt^. *p <0.05, Friedman test. N = 3 pancreases. **(E)** Representative confocal microscopy immunostaining of HPSs after 24 hours of 300 nM miR-30d-5p inhibitor transfection (coupled with FAM, green), insulin in white and DAPI in blue. Scale: 50 μm. Green bars on the right represent the counts of miR-30d-5p inhibitor with respect to beta cells (insulin positivity) in the different concentrations used (n=3 different images for each condition). Statistical significance by Kruskal–Wallis with Dunn’s *post-hoc* test. **(F)** Left, dynamic insulin secretion in response to glucose after 8 days of miR-30d-5p inhibition (red line), control inhibitor (blue line) or untreated (green) in three different pancreases. Perifusion was made during 90 min by using high glucose (16.7 mM) and low glucose (1 or 3 mM) stimulation. X-axis: time/[Glucose]; Y-axis: Insulin secretion (mU/L) normalized to KCL. Data are mean and error. N = 6 slices in the perifusion chamber for each treatment condition. Right, stimulation index normalized by KCl during the high glucose stimulation period (G16.7). Results are expressed as mean ± SEM. Statistics were calculated by unpaired two-tailed t-test (∗∗∗p < 0.001; ∗∗∗∗p < 0.0001), with individual pancreatic slices analyzed as experimental units across three independent donor pancreases.

To study pancreatic cell function and processes consistent with the emergence of insulin-expressing cells in a context that preserves native pancreatic architecture and cell–cell interactions without beta cell dedifferentiation processes, HPSs were used. As current publications on the transfection of HPSs with miRNAs are lacking, a protocol was developed to efficiently deliver miR-30d-5p inhibitor or mimic probes to human beta cells (**Figure 1A**). Briefly, HPSs are obtained by vibratome slicing and cultured for up to 10 days in AirHive dishes. Processes related to beta cell proliferation and differentiation can be assessed after transduction with reporter and lineage tracer adenoviruses, which allows tracking of beta cell proliferation and differentiation over time (26, 27). Pancreatic slices from eight non-diabetic donors were transfected with lipid-based reagents carrying specific oligonucleotides to evaluate the functional and regenerative effects of miR-30d-5p on beta cells.

First, we confirmed the expression of miR-30d-5p in five different pancreases (**Figure 1B**). Then, in one of them, we performed a time-course experiment to determine the concentration at which optimal inhibition of miR-30d-5p was achieved and to assess the necessity of retransfection to maintain inhibition over time, given that the slices would be maintained for up to 10 days in culture. We observed that a single transfection with all tested concentrations (200, 300 and 400 nM) was sufficient to maintain the inhibition of miR-30d-5p (~10,000-fold reduction) up to 7 days later (from 24 to 168h). Therefore, re-transfection was not deemed necessary (**Supplementary Figure 1A**). As recovery of miR-30d-5p expression was observed on day 7 with a concentration of 200 nM, we decided to perform the next experiments with 300 nM of miR-30d-5p inhibitor and 200 nM of miR-30d-5p mimic so as not to saturate the miRNA-processing cell machinery.

72 hours following the transfection of three pancreases with either the inhibitor, control inhibitor, mimic, or control mimic, we confirmed the successful inhibition of endogenous miR-30d-5p in comparison to the basal condition (**Figure 1C**). No significant increase in miR-30d-5p levels was observed following the application of the mimic. As anticipated, transfection with the inhibitor and mimic controls did not result in notable alterations in miRNA expression, which confirms the specificity of the probes. Moreover, to further assess the impact of miR-30d-5p inhibition, we analyzed the expression of one of its target genes, *IRS*. As expected, a significant increase in the expression of this gene can be detected after the inhibition of miR-30d-5p (**Figure 1D**). 24 hours after transfection, the presence of miRNA inhibitor in human pancreatic tissue, and specifically in beta cells, was also analyzed by immunostaining. As expected, the elevated inhibitor concentration resulted in a heightened signal, both in terms of the total number of cells (as observed through nuclear staining with DAPI, indicated in blue) and the number of beta cells (as evidenced by insulin staining, indicated in white) (**Figure 1E**). No signal was detected in untransfected slices (basal). Furthermore, confocal microscopy images demonstrate the colocalization of insulin and miR-30d-5p inhibitor within the same beta cell, indicating that the inhibitor is being transfected into the cells of interest. Furthermore, a fluorescence-based assay was conducted to detect mitochondria in live cells, with the aim of determining tissue viability 24 and 168 hours after transfection. The results showed good tissue viability in both cases (**Supplementary Figure 1B**).

After confirming the correct delivery and effect of miR-30d-5p inhibitor on HPSs, we next assessed beta cell function using a dynamic perifusion system to accurately replicate the physiological secretion of insulin (23). HPSs were maintained in complete medium (non-treated) or were transfected with 300 nM of miR-30d-5p inhibitor or control inhibitor 8 days prior to perifusion. The viability of the slices was good after 10 days of culture (**Supplementary Figure 1C**). We observed that the inhibition of miR-30d-5p in three different pancreases correlates with a significant decrease in first-phase C-peptide secretion after glucose stimulation. Indeed, stimulation index at 16.7 mM glucose stimulation phase was statistically reduced by miR-30d-5p inhibitor when compared to inhibitor control (P = 0.0003) or untreated slices (P<0.0001) (**Figure 1F**). Altogether, our results indicate that miR-30d-5p downregulation contributes to impaired beta cell function in HPSs.

### miR-30d-5p overexpression is associated with the emergence of insulin-expressing cells

**Figure 2:**
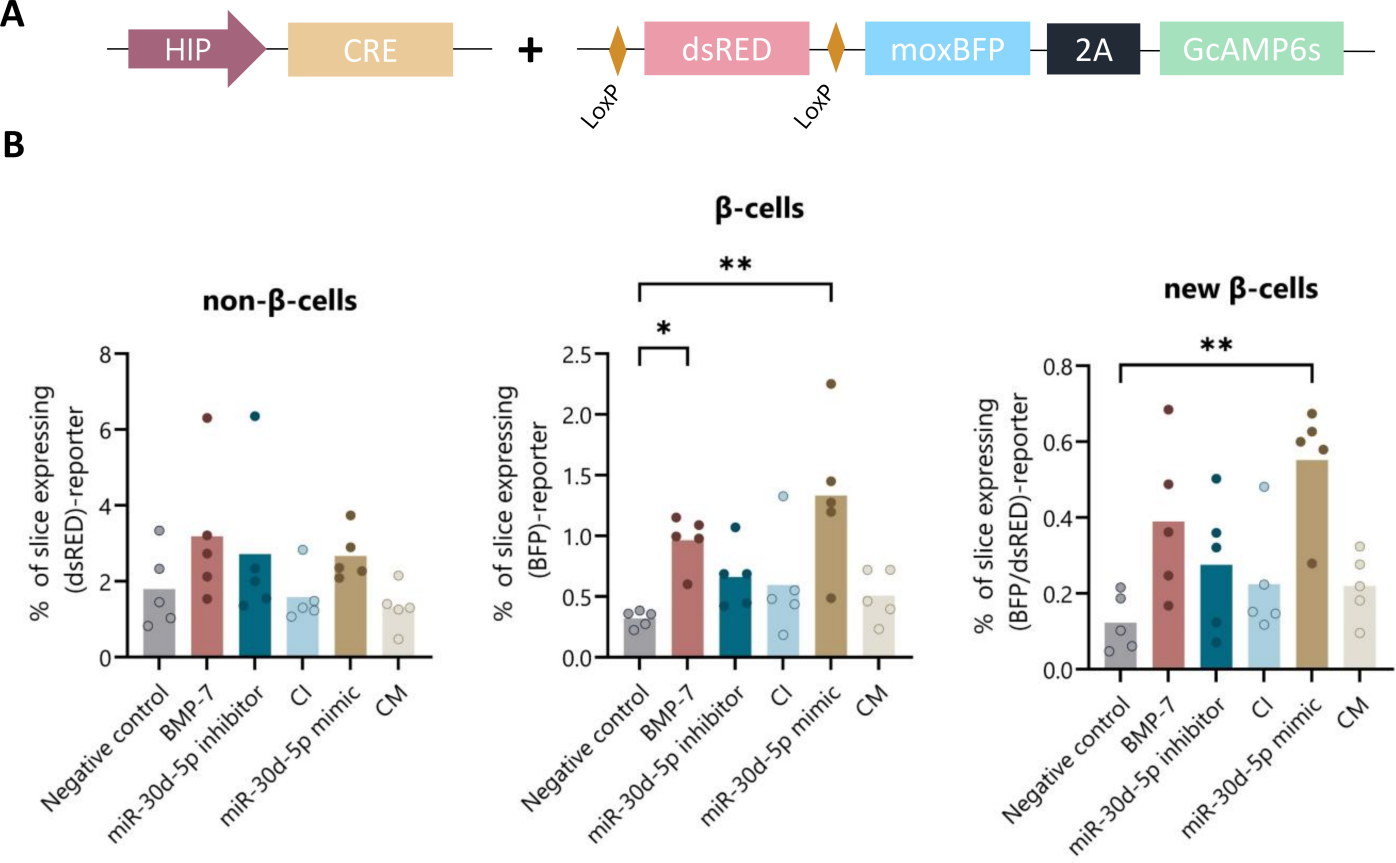
miR-30d-5p mimic is associated with changes suggestive of insulin-expressing cell emergence in human pancreatic slices. **(A)** Reporter strategy involving the co-transduction with an adenoviral reporter construct CMV-loxP-dsRED-loxP-BFP-2A-GCaMP6 and a HIP-driven Cre recombinase construct. **(B)** Quantification of dsRED (non-beta cells), BFP (beta cells) and dsRED+BFP (new insulin-producing cells) expression across the different treatment groups. Between 1 and 3 regions of interest were analyzed for each condition of a pancreas (n=2 slices per condition) and the mean was generated. Data are presented as mean and each dot represents the mean of the pancreases analyzed on one day from D6 to D10 (n=2 pancreases). ns ≥0.05, *p <0.05, and **p <0.01 by Kruskal–Wallis with Dunn’s *post-hoc* test. CI, control inhibitor; CM, control mimic.

Qadir et al. established proof of principle that an extended culture of HPSs could be used to study regeneration, and that treatment with BMP receptor agonists such as BMP-7 results in the appearance of new insulin-producing beta cells in pancreatic slices (26). Based on this study, slices were generated from two deceased non-diabetic donors and co-transduced with adenoviruses carrying the reporter construct CMV-*loxP*-dsRED-*loxP*-BFP-2A-GCaMP6 and a HIP-driven Cre recombinase (**Figure 2A**). As reported, the prediction was that doubly transduced non-beta cells would be tagged red and pre-existing beta cells would be tagged blue. Moreover, if a beta-cell differentiates from a non-beta-cell, there is a window of time during which BFP is already detected but the dsRED protein (no longer expressed) has not been degraded yet. Hence, the transient violet (red+blue) color of these cells can be used to identify new insulin-producing beta cells (**Supplementary Figure 1D**). Following viral transduction, slices were transfected with miR-30d-5p inhibitor/mimic or their respective controls to analyze the effect of miRNA modulation on the emergence of insulin-expressing cells. As a positive control, slices were treated with 100 pg/mL BMP-7 for 5 days, followed by withdrawal for 5 days, as reported (26, 27).

The quantification of the fluorescent signals on specific regions of interest from day 6 to day 10 (n=2 slices/condition) on the two pancreases confirmed a higher percentage of the slices expressing the blue signal in those treated with BMP-7 or miR-30d-5p mimic in comparison to the untreated slices (**Figure 2B**). A significant increase in the slice expressing the violet signal (red+blue) was observed only in those treated with the miR-30d-5p mimic compared to the untreated slices, indicating that upregulation of this miRNA may contribute to the formation of new insulin-producing cells (**Supplementary Figure 1D**). No statistically significant differences were found between the other treatment groups.

### miR-30d-5p modulation is associated with gene expression changes in pancreatic slices related to pathways involved in beta cell function, survival and regeneration

Bulk RNA-seq was used to evaluate transcriptional changes following miR-30d-5p overexpression or inhibition in pancreatic cells, using two HPSs per condition from three donor pancreases (the ones used in **Figure 1C**). Since RNA was extracted from whole HPSs, the resulting transcriptomic changes reflect the combined contribution of multiple pancreatic cell populations, including endocrine (around 1-2%), exocrine (major fraction, 80-90%), stromal, and immune cells, and thus cannot be specifically attributed to beta cells. We observed that after accounting for donor variation by performing a ComBat correction with the donor as a covariate, there were no differences between control groups (**Supplementary Figure 2A**). Therefore, in order to facilitate comparisons between experimental treatments, the mimic and inhibitor controls were treated as a single group. Differential expression analysis revealed 273 DEGs for the inhibitor compared with the controls (66 upregulated, 207 downregulated; **Supplementary Figure 2B**), 518 DEGs for the mimic compared with the controls (231 up, 287 down; **Supplementary Figure 2C**), and 480 when comparing the inhibitor with the mimic (209 up, 271 down; **Supplementary Figure 2D**). The top upregulated DEGs after miR-30d-5p silencing included genes such as *GLUT6* (glycolysis), *PRG2* (immune response), *TGFB3* (cell proliferation and differentiation), or *CYP4F8* (fatty acid biosynthesis), while downregulated genes such as *UTP14C* (cell cycle regulation), *KDM6A* (catabolic processes), *MRPL15* (mitochondrial protein synthesis) or *DLK1* (cell growth). The top upregulated DEGs after miR-30d-5p upregulation included genes such as *HOOK1* (endocytosis), *A2ML1* (inhibition of proteases), *CDH8* (cell-cell adhesion), or *KCNH2* (potassium channel activity), and decreased expression of *RB1* (negative regulation of the cell cycle), *PRDM10* (cell growth), *MED12* (regulation of gene activity), and *STAT5B* (signal transduction of cytokines). Compared to the mimic, the inhibitor upregulated genes such as *FAS*, *PRDM10*, *RAB19*, or *CDCA4*, which are associated with apoptosis, growth, autophagy, and proliferation.

**Figure 3:**
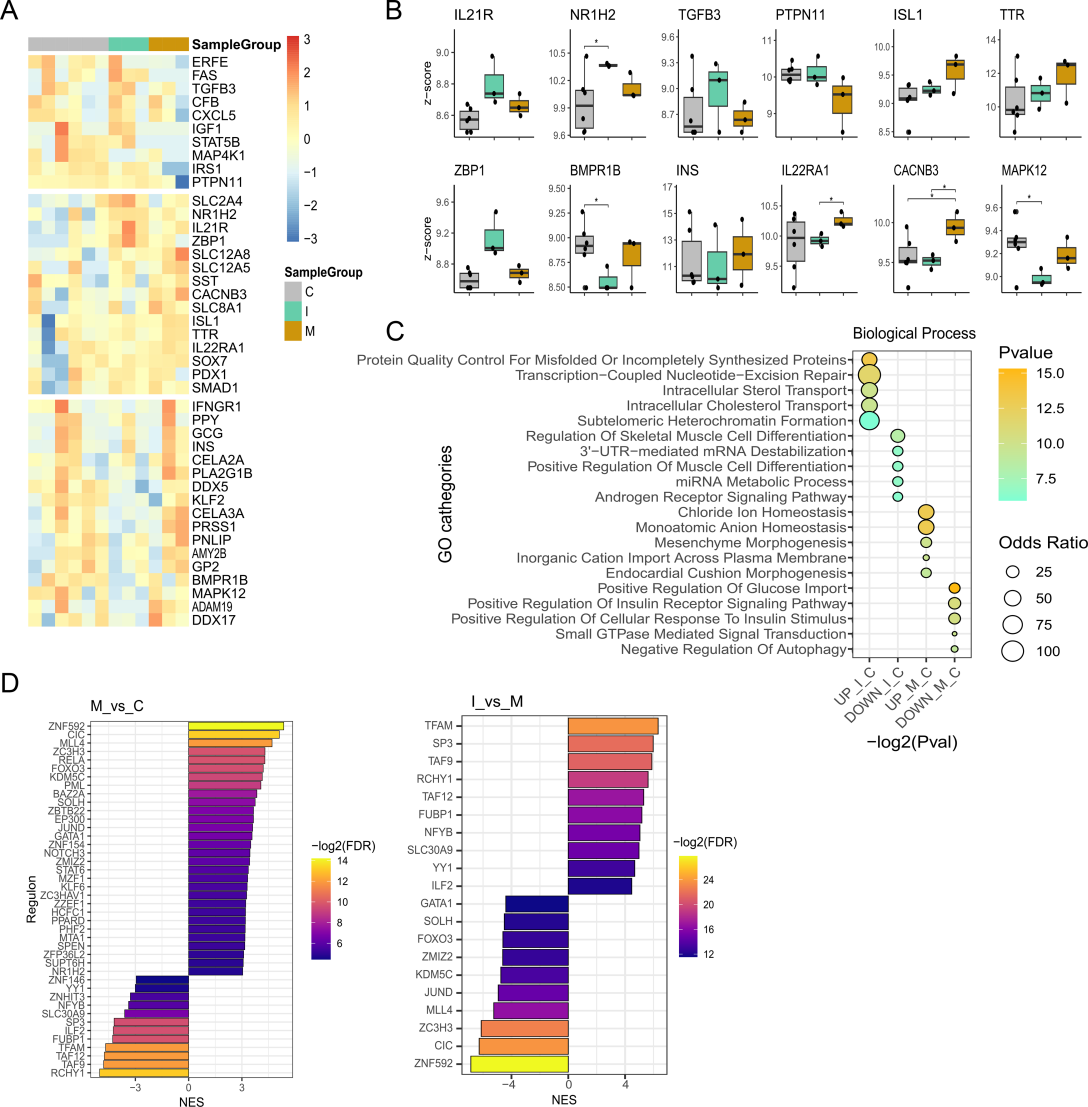
Selected gene expression pattern after miR-30d-5p modulation. **(A)** Hierarchical clustering heatmap showing selected genes with significantly different expression levels (DEGs) between the control group (gray, comprising both control mimic and control inhibitor), the inhibitor (green) and the mimic (orange). Each column represents individual samples (treated slices from 3 pancreases), and each row represents an individual gene. Log (FC) >0.5 for upregulated DEGs (red), and log (FC) <0.5 for downregulated DEGs (blue); FDR adjusted p-value ≤0.05. **(B)** Boxplots of the normalized expression (z-score) of some genes from the bulk RNA-seq data in controls (gray), miR-30d-5p inhibitor (blue), and miR-30d-5p mimic (orange). Error bars indicate 95% confidence intervals and the center line is the median. N = 3 pancreases (ns ≥0.05, *p <0.05, by t-test). **(C)** Gene ontology (GO) overrepresentation of GO Biological Process categories comprising the upregulated and downregulated DEGs in comparison to the control group after miR-30d-5p functional silencing or upregulation. The odds ratios for each group and the −log2(p-value) are shown. Selected significant categories (p-value ≤0.05) are shown. **(D)** Discriminant Regulon Expression Analysis (DoRothEA) of miR-30d-5p mimic vs. control (left) and miR-30d-5p inhibitor vs. mimic (right). Normalized enrichment score (NES) and -log2(FDR adjusted p-value) of transcription factor expression are depicted.

To explore transcriptional changes in pancreatic slices related to pathways involved in endocrine function, cell survival, and immune responses, we selected key DEGs to compare across groups (**Figure 3A**). The heatmap revealed three clusters of DEGs. The first cluster includes genes downregulated by the miR-30d-5p mimic, such as *FAS*, *ERFE*, *STAT5B*, *IRS1*, *IGF1*, or *MAP4K1* (**Figure 3A**). Notably, *IGF-1* and *IRS-1* are part of the insulin signaling pathway, which appears to be modulated in pancreatic slices by the miRNA. Indeed, we validated *IRS-1* as one of its target genes, since in the presence of the inhibitor, the expression of *IRS-1* increases (**Figure 1D**). Despite this, miR-30d-5p mimic slightly increased insulin gene (*INS*) expression compared to controls and inhibitor (**Figure 3B**), possibly reflecting insulin autocrine negative feedback. The second cluster shows genes upregulated by both mimic and inhibitor in comparison to controls, including *IL21R*, *NR1H2*, *SLC12A8*, or *SLC12A5*, with clear differences between treatments. For example, inhibition of miR-30d-5p leads to a significant increase in the expression of *ZBP1* —a potent innate immune sensor, *IL21R* —which helps to maintain an inflammatory environment, or *NR1H2* —which encodes for liver X receptor β (LXR-β), whose activation can impair insulin secretion (28). Conversely, the mimic upregulates genes like *IL22RA1*, *TTR*, *ISL1*, or *CACNB3* compared to the controls and the inhibitor. In particular, *ISL-1* supports pancreatic endocrine function and survival, *TTR* promotes insulin secretion, and *CACNB3* modulates further insulin secretion by reducing Ca²^+^ oscillations. The third cluster includes genes downregulated by miR-30d-5p inhibition compared to the controls and mimic groups, such as *MAPK12* (stress response), *BMPR1B* and *KLF2* (pancreatic progenitor differentiation), *INS*, and *CELA3A* (pancreatic elastase) (**Figure 3B**).

The GO analysis revealed no overlap between biological processes enriched in up- or downregulated genes after miR-30d-5p modulation (**Figure 3C**). After miRNA silencing, functional categories such as quality control of misfolded or incompletely synthesized proteins and intracellular transport of sterols and cholesterol were enriched among the set of upregulated genes. In contrast, functional categories such as regulation of muscle cell differentiation, the androgen receptor pathway, and miRNA metabolic process were enriched among the set of deregulated genes. Therefore, the absence of miR-30d-5p could be related to cellular stress and altered pancreatic cell survival, proliferation, and insulin secretion pathways, caused by misfolded proteins and a lack of cell differentiation and signaling processes through the androgen receptor, which can activate various signaling pathways, including PI3K/AKT (29). After miR-30d-5p mimic treatment, upregulated genes were enriched in chloride ion and anion homeostasis, cation import, and mesenchyme morphogenesis—processes linked to electrical activity for insulin secretion and cell differentiation. Conversely, downregulated genes were enriched in insulin response regulation and glucose import, once more reflecting the negative feedback signal of insulin within the islet (**Figure 3C**). Subsequently, we examined transcription factors (TFs) involved in the transcriptomic changes after miR-30d-5p modulation. Despite the inability to identify any enriched TF following miR-30d-5p silencing in comparison to the control group, the upregulated genes following miR-30d-5p mimic treatment exhibited associations with *FOXO3, NOTCH3, JUND*, and *NR1H2*, which have been linked to beta cell proliferation, survival, and function (**Figure 3D**). A comparison of the regulon of the inhibitor versus the mimic revealed that *JUND* and *FOXO3* were associated with downregulated genes. In summary, modulation of miR-30d-5p influences genes involved in insulin synthesis, pancreatic cell survival and proliferation, and immune activation.

### *In vivo* upregulation of miR-30d-5p influences autoimmune diabetes progression and immune cell composition

**Figure 4:**
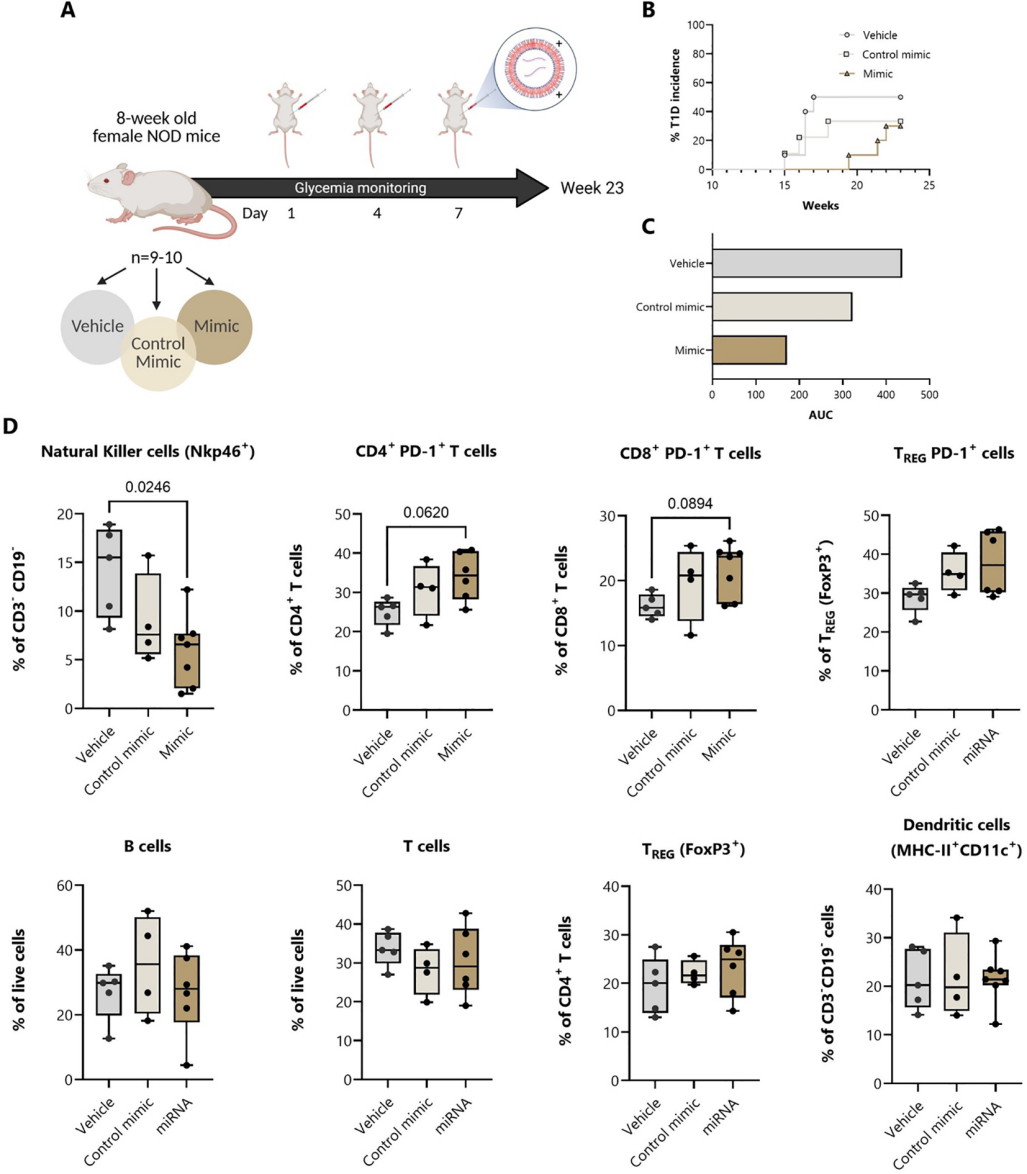
Effect of miR-30d-5p mimic on autoimmune diabetes incidence in NOD mice. **(A)** Schematic representation of the mouse treatment protocol. Three groups of at least nine 8-week-old prediabetic female NOD mice were treated with vehicle, control mimic, or miR-30d-5p mimic. Mice received 3 intraperitoneal doses every three days of 200 μL 5% glucose solution containing 80 μg *in vivo* mimic or control mimic (0.4 μg/μL per injection) and *in vivo*-jetPEI at N/P=8. Glycemia levels were monitored until the end of the follow-up (23 weeks) to determine diabetes incidence. **(B)** Cumulative incidence (percentage) of autoimmune diabetes in NOD mice treated with vehicle (gray, n=10), control mimic (light brown, n=9) and miR-30d-5p mimic (dark brown, n=10). No significant differences in autoimmune diabetes incidence were found between groups (Mantel-Cox Log-Rank). **(C)** Area under the curve (AUC) of the cumulative incidence. **(D)** Percentage of Natural Killer cells, PD-1^+^CD4^+^ T cells, PD-1^+^CD8^+^ T cells, T_REG_ PD-1^+^ cells, B cells, T cells, T_REG_ FoxP3^+^, and dendritic cells after treatment with vehicle (gray, n=5), control mimic (light brown, n=4) and miR-30d-5p mimic (dark brown, n=7). Data are presented as box-and-whisker plots. Boxes indicate the first and third quartiles. The horizontal bar in the box indicates the median. Each dot shows a single sample (p <0.05, Kruskal Wallis and Dunn’s test).

Having established the effects of miR-30d-5p on HPSs, we next assessed the impact of miR-30d-5p on autoimmune diabetes progression using the NOD mouse. Three groups of at least 9 normoglycemic, 8-week-old NOD mice were treated with 1) miRNA mmu-miR-30d-5p, 2) control mimic, or 3) vehicle (jetPEI) as shown in **Figure 4A**. Remarkably, NOD mice receiving three i.p. injections of miR-30d-5p mimic developed autoimmune diabetes later than those given a control mimic or vehicle (**Figure 4B**). The delay was 4.6 weeks when compared to control mimic-treated mice, and 4.9 weeks compared to vehicle-treated mice. At the end of the follow-up period, type 1 diabetes incidence tended to be lower in the miRNA mimic-treated group (30%) compared with the vehicle-treated group (50%), although without reaching statistical significance. This result is reflected in the AUC (**Figure 4C**). However, the control mimic group also exhibited a reduced incidence of autoimmune diabetes (33%), suggesting a non-specific effect of the treatment. The treatment with miR-30d-5p mimic quantitatively altered the leukocyte subsets in the spleen at the end of the follow-up, reducing the percentage of Natural Killer (NK) cells and tending to increase the percentage of PD-1^+^CD4^+^ and PD-1^+^CD8^+^ T cells (**Figure 4D**). Gating strategy is shown in **Supplementary Figure 3**. These findings highlight the long-term immunomodulatory effect of miR-30d-5p on autoimmune diabetes, which may be associated with a delayed disease course, potentially linked to PD-1–mediated T-cell exhaustion and reduced effector activity over time.

### miR-30d-5p influences the expression of immunoregulatory molecules in human T lymphocytes

To assess the impact of miR-30d-5p inhibition on T cells, we first confirmed that these cells express the desired miRNA and compared its expression levels between different T-cell subpopulations. For that, we sorted cytotoxic CD8, conventional CD4 and Tregs from pediatric patients during PR or non-PR, since the expression levels of miR-30d-5p in plasma were upregulated in remitter patients. It was observed that both CD4 and CD8 lymphocytes from remitters and non-remitters expressed significantly higher levels of miR-30d-5p compared to Tregs (**Supplementary Figure 4A**). For each T cell subset, the expression profiles between remitter and non-remitter patients were comparable, suggesting that T cells are not the source of the previously found differences. After confirming the expression of miR-30d-5p in T cells, we next assessed the impact of miR-30d-5p inhibition on their phenotype and function. To this end, we activated and expanded CD3^+^ T cells *in vitro* from seven newly diagnosed type 1 diabetes children and six age- and sex-matched controls (see **Supplementary Table 4** for details of clinical and metabolic data).

**Figure 5:**
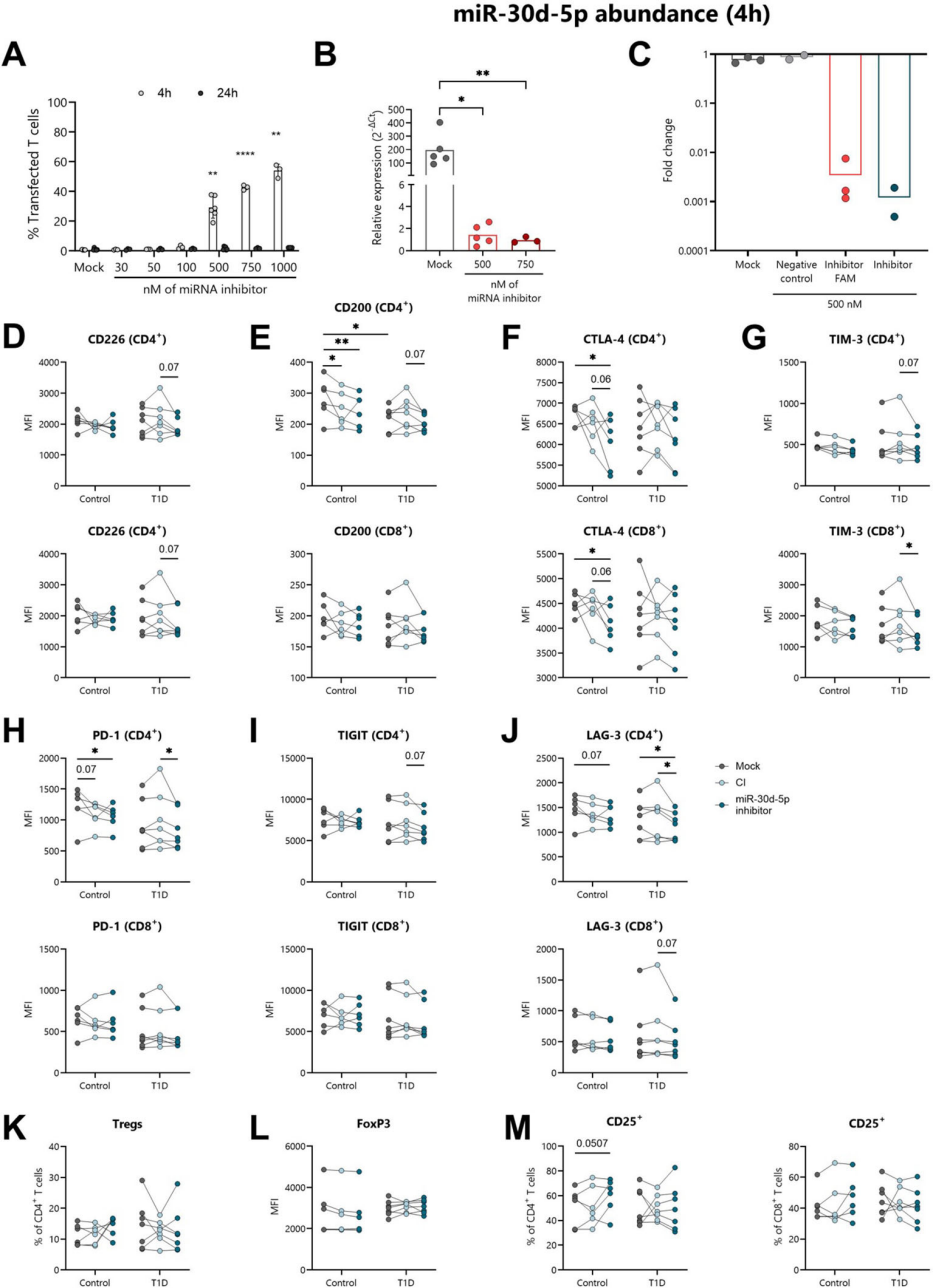
miR-30d-5p inhibitor’s delivery to human T lymphocytes decreases the expression of inhibitory molecules on both CD4^+^ and CD8^+^ subsets. **(A)** Percentage of transfected T cells 4 and 24 hours after electroporation with different concentrations of miR-30d-5p inhibitor conjugated with FAM. **p < 0.01, and ****p < 0.0001 Mixed-effects analysis with Tukey’s multiple comparisons test (n=3–6). **(B)** Relative expression of miR-30d-5p analyzed by single-assay RT-qPCR 4 hours after electroporation with 500 or 750 nM of miRNA inhibitor coupled with FAM (n=3–5). The miRNA expression signal was normalized to 5S rRNA expression. Values are expressed as 2^-ΔCt^. *p ≤0.05, **p ≤0.01, Kruskal–Wallis with Dunn’s *post hoc* test. **(C)** Fold change in the miR-30d-5p expression after 500 nM of miR-30d-5p inhibitor (red, conjugated with FAM; blue, uncoupled) or inhibitor control (light gray) using basal transcription (complete medium-cultured T cells) as standard value (n=2–3). The miRNA expression signal was normalized to 5S rRNA expression. Values are expressed as 2^-ΔΔCt^. In all cases, a mock transfection was used as a negative control (cells electroporated without the oligonucleotide). **(D–J)** Mean fluorescence intensity (MFI) of CD226, CD200, CTLA-4, TIM-3, PD-1, TIGIT, and LAG-3 expression in CD4^+^ (up) and CD8^+^ (down) T lymphocytes obtained from type 1 diabetes (T1D) at diagnosis (n=7) and control (n=6) subjects. From D to J, within each group, gray dots represent T lymphocytes simply electroporated (mock), light blue dots represent T cells electroporated with a control inhibitor (CI) and dark blue dots with a miR-30d-5p inhibitor (500 nM). **(K)** Percentage of Tregs in CD4^+^ T lymphocytes from T1D at diagnosis (n=7) and control (n=6) subjects. **(L)** Mean fluorescence intensity (MFI) of FoxP3 in CD4^+^ T cells from T1D at diagnosis (n=7) and control (n=6) subjects. **(M)** Percentage of CD25^+^ cells in CD4^+^ and CD8^+^ T cells from T1D at diagnosis (n=7) and control (n=6) subjects. Statistical analysis was performed using repeated-measures two-way ANOVA with Geisser–Greenhouse correction, followed by Tukey’s *post hoc* test. *p < 0.05, and **p < 0.01.

The delivery of the miR-30d-5p inhibitor was conducted by T-cell electroporation. First, concentrations from 30 to 1,000 nM of 6-carboxyfluorescein (FAM)-labeled inhibitor were tested to determine the minimum effective concentration without impacting cell viability, which remained consistently above 90% across all conditions. Transfection efficiency increased with concentration, exceeding 20% only at ≥500 nM. Transfected FAM-positive cells were detectable 4 h post-electroporation but not at 24 h (**Figure 5A**), most likely due to the rapid proliferation of activated T lymphocytes (**Supplementary Figure 4B**). To assess functional impact, 500 and 750 nM of the FAM-conjugated inhibitor were tested on T cells, both showing strong miR-30d-5p suppression versus mock condition 4 h post-electroporation (**Figure 5B**). Since 500 nM proved effective, it was used in all subsequent assays. Electroporation with 500 nM of either conjugated or unconjugated inhibitor induced up to a 1,000-fold reduction in miR-30d-5p levels, while mock and negative controls had no effect (**Figure 5C**). It should be noted that differences between fluorescently assessed transfection efficiency and RT-qPCR-based miR-30d-5p suppression may reflect heterogeneous uptake across T cell subsets, differences in assay sensitivity between the two techniques, and preferential transfection of specific cell subsets, and should be considered when interpreting the magnitude of the observed effects.

The analysis of immune checkpoints or co-inhibitory receptors (LAG-3, TIM-3, PD-1, TIGIT, CTLA-4), a co-inhibitory ligand (CD200) and a co-stimulatory receptor (CD226) on T lymphocytes was performed 24 h after miR-30d-5p blockade. Gating strategy is shown in **Supplementary Figure 5**. The analysis revealed a general decrease in the expression of the inhibitory molecules both on CD4^+^ and CD8^+^ T cells. A representative analysis of these markers is shown in **Supplementary Figure 6**. A tendency for a reduced CD226 expression in the absence of the miRNA in both CD4^+^ and CD8^+^ T cells was observed in comparison to cells treated with the control inhibitor probe (**Figure 5D**). MiR-30d-5p inhibition reduced CD200, LAG-3, and PD-1 expression on CD4^+^ T cells in both controls and type 1 diabetes patients in comparison to both negative controls (mock and control inhibitor probe) (**Figures 5E, H, J**). CTLA-4 decreased in controls (**Figure 5F**), while TIGIT and TIM-3 tended to be reduced in type 1 diabetes patients in the absence of miR-30d-5p 3 (**Figures 5G, I**). CD200 expression in control subjects was also lower in T cells electroporated with the control inhibitor probe in comparison to the mock condition, suggesting off-target effects. Moreover, patients with type 1 diabetes exhibited a lower expression of CD200 than controls when comparing mock conditions (**Figure 5E**). This observation may reflect a deficiency in the expression of this molecule in patients and a reduced ability to suppress immune responses. In CD8^+^ T cells, miR-30d-5p blockade reduced TIM-3 in type 1 diabetes and CTLA-4 in controls, with slight decreases in LAG-3 also observed in type 1 diabetes (**Figures 5F, G, J**). No significant differences were observed in Tregs percentage, and CD25 and FoxP3 expression levels in CD4^+^ and CD8^+^ T cells between groups (**Figures 5K–M**). Overall, these results indicate that miR-30d-5p contributes to the regulation of several inhibitory pathways in T cells, and its inhibition results in a decrease in suppressive markers consistent with a more activated T-cell phenotype.

The percentage of CD4^+^ and CD8^+^ T cells expressing these molecules was also analyzed. Overall, changes in frequencies largely mirrored those observed for MFI. In control subjects, miR-30d-5p inhibition reduced the proportion of CD4^+^ and CD8^+^ T cells expressing several inhibitory receptors, including CD200, CTLA-4, PD-1, TIM-3, and LAG-3 (**Supplementary Figures 4D–I**). In type 1 diabetes patients, similar trends were observed, with decreased frequencies of PD-1^+^ and LAG-3^+^ CD4^+^ T cells and reduced LAG-3 and PD-1 expression in CD8^+^ T cells under miR-30d-5p blockade. No differences were detected in CD226^+^ or TIGIT^+^ T cell subsets (**Supplementary Figures 4C, H**).

We also investigated whether the absence of miR-30d-5p could influence the maturation, activation and differentiation of T lymphocytes into Tregs. For Treg identification, FoxP3 was used as the defining marker, since membrane CD25 expression is strongly upregulated upon T cell activation and therefore is not suitable to reliably distinguish Tregs under these conditions. Concerning CD45RA and CCR7 expression, markers used to identify naïve or memory populations, we observed a modest increase in the proportion of CD4^+^CD45RA^+^CCR7^+^ (naïve) and CD8^+^CD45RA^+^CCR7^-^ (Terminally Differentiated Effector Memory, TEMRA) T cells, together with a decline in CD4^+^CD45RA^-^CCR7^-^ (effector memory) T cells when miR-30d-5p was suppressed (**Supplementary Figure 7A**). No significant differences were observed in the percentage of CD4^+^ and CD8^+^ T cells expressing the activation marker CD69, nor in the percentage of activated (CD69^+^) Tregs (**Supplementary Figures 7C, D**). Therefore, the absence of miR-30d-5p is associated with an increase in CD45RA-expressing subsets.

### miR-30d-5p inhibition alters IFN-γ secretion but does not impair T cell proliferation

**Figure 6:**
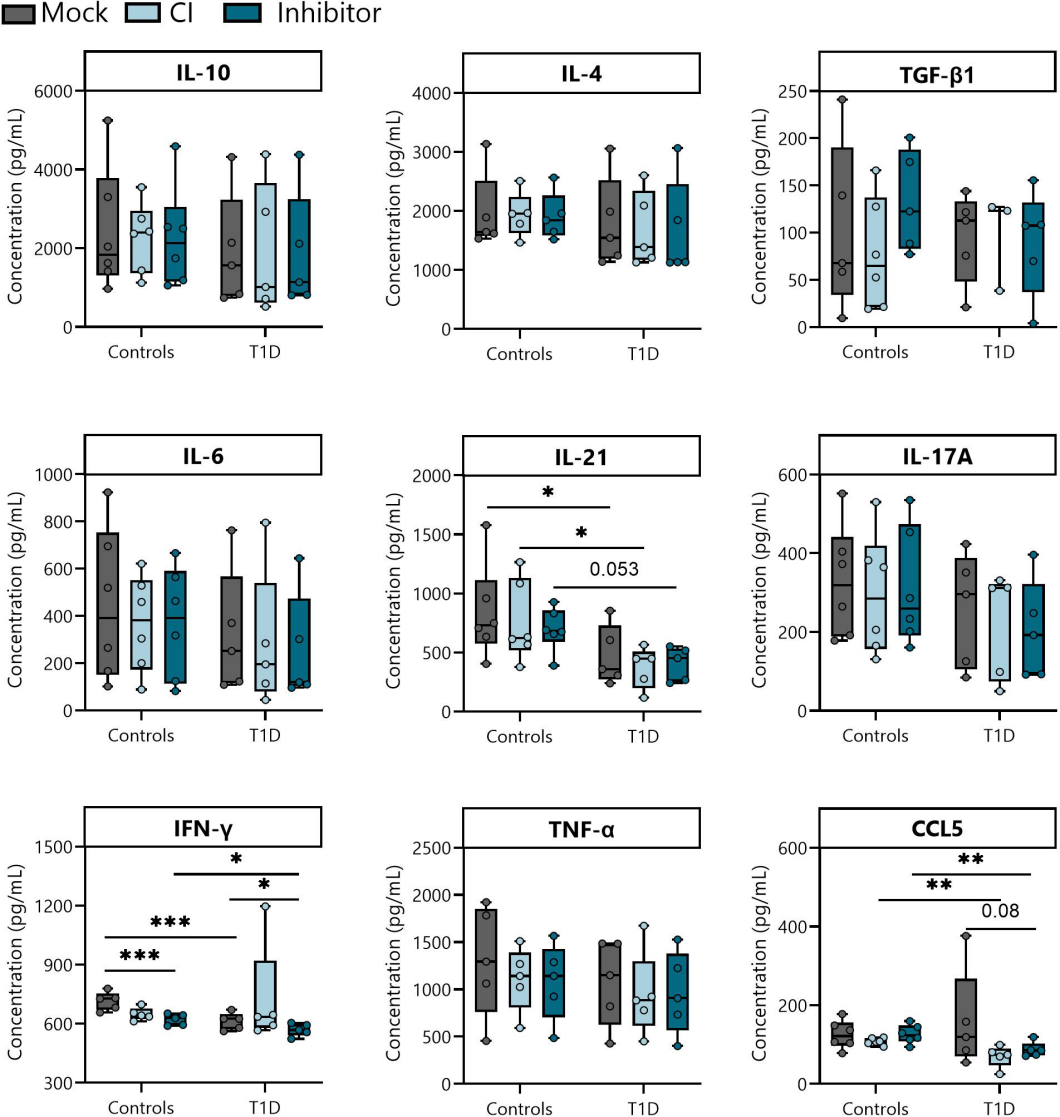
Functional effect of miR-30d-5p inhibition on the cytokine profile of T lymphocytes. Concentration of cytokines IL-10, IL-4, TGF-β1, IL-6, IL-21, IL-17A, IFN-γ and TNF-α and chemokine CCL5 in the supernatant of re-stimulated T lymphocytes for 2 days with IL-2 and antibodies anti-CD3/CD28/CD2 obtained from control subjects (n≥5), and patients with type 1 diabetes (n≥3). Within each group, gray boxes represent T lymphocytes simply electroporated (mock), light blue boxes represent T cells electroporated with a control inhibitor (CI) and dark blue boxes with a miR-30d-5p inhibitor. Data are presented as box-and-whisker plots. Boxes indicate the first and third quartiles. The horizontal bar in the box indicates the median. Each dot shows a single sample. ns ≥0.05, *p <0.05, **p <0.01, ***p <0.001 repeated measures two-way ANOVA with the Geisser-Greenhouse correction and Tukey’s multiple comparisons test.

Twenty-four hours after electroporation, T cells were cultured for two more days in medium alone, or re-stimulated with IL-2, or with anti-CD3/CD28/CD2 plus IL-2 to evaluate both proliferative capacity and cytokine secretion. Proliferation did not differ significantly across electroporation groups irrespective of the stimulation applied (**Supplementary Figure 7E**), indicating that miR-30d-5p inhibition does not impair T-cell proliferative responses. Cytokine secretion profiles revealed overall stability across conditions, with most cytokines remaining unchanged. However, a consistent reduction in IFN-γ levels was observed after inhibition of miR-30d-5p compared to the mock condition, both in type 1 diabetes patients and control subjects (**Figure 6**). These findings indicate that while electroporation and miRNA modulation do not globally disrupt T-cell function, inhibition of miR-30d-5p leads to reduced IFN-γ production. When comparing disease groups, T cells from type 1 diabetes patients secreted markedly lower amounts of IFN-γ, IL-21, and CCL5 than those from healthy controls, suggesting an impaired proinflammatory and helper T-cell cytokine profile. In contrast, secretion of IL-10, IL-4, IL-6, IL-17A, TNF-α, and TGF-β1 did not show significant differences between type 1 diabetes and control subjects.

## Discussion

MiRNAs have emerged as key post-transcriptional regulators of immune responses and beta cell function, positioning them as potential modulators of disease progression in type 1 diabetes. In this study, we describe the immunoregulatory potential of miR-30d-5p in association with changes in beta cell autoimmunity and functional recovery. Importantly, our findings provide a framework to interpret the PR phase not merely as a transient clinical phenomenon, but as an active, regulated state potentially influenced in part by epigenetic factors such as miRNAs. The upregulation and functional effects of miR-30d-5p suggest that it may contribute to the coordinated changes in immune tolerance and beta cell function observed during PR. In this context, miR-30d-5p may represent both a biomarker and a putative mediator of this phase, linking metabolic recovery with immune regulation. These results support the concept of PR as a critical therapeutic window, during which modulation of key regulators like miR-30d-5p could potentially help sustain beta cell function and influence disease trajectory.

In addition to its immunoregulatory effects, miR-30d-5p appears to play a role in beta cell regeneration and function. Using lineage tracing in HPSs, we found that miR-30d-5p upregulation correlated with the appearance of new insulin-producing cells, surpassing even BMP-7, a known positive control (27, 30). Although these data are consistent with the emergence of insulin-positive cell formation, we recognize that additional mechanisms, including insulin re-expression or α-to-β cell conversion, may also contribute and cannot be formally distinguished without more comprehensive lineage tracing analyses. While this supports a potential novel role for miR-30d-5p in beta cell regeneration, caution is needed, as excessive overexpression in transgenic models has been linked to beta cell dedifferentiation (31). Other miRNAs, such as miR-132, miR-375, miR-23a, and miR-7 have also been implicated in beta cell regeneration through diverse mechanisms (32–35). Regarding functionality, it has been demonstrated that miR-30d activates MafA, a beta cell-specific transcription factor, and insulin production by targeting TNF-α-activated *MAP4K4* in MIN6 cells and islets (14, 15, 36). Using a perifusion system to mimic *in vivo* conditions, we observed that inhibiting miR-30d-5p in HPSs was associated with reduced insulin secretion in response to high glucose, consistent with prior results. Thus, metabolic improvement during the PR phase may reflect both recovery of existing beta cells and processes associated with new insulin-producing cells formation following increased miR-30d-5p levels. On the other hand, RNA-seq analysis revealed that miR-30d-5p upregulation after mimic treatment was associated with altered expression of genes related to beta cell function. *INS* expression tended to increase after 72 hours of transfection with the mimic, along with modest upregulation of *TTR* and *ISL1*, both supporting insulin secretion and beta cell function. miR-30d-5p mimic treatment was associated with modulation of calcium-handling genes, including increased expression of *KCNH2*, which encodes a potassium channel that regulates insulin secretion and limits intracellular Ca²^+^ influx, and *CACNB3*, which modulates Ca²^+^ influx and oscillations. Indeed, an immediate effect of insulin secretion can initiate a negative feedback loop that regulates and prevents the overproduction of insulin (37). On the other hand, the inhibition of miR-30d-5p upregulated *NR1H2* (LXR-β), known to suppress insulin secretion (38). Beyond secretion, miR-30d-5p was associated with changes in cell signaling pathways. Silencing it reduced BMP pathway genes (*BMPR1B, KLF2*) and increased *TGFB3*, findings consistent with possible modulation of BMP and TGF-β signaling. BMP signaling is required for the expression of beta cell genes such as *PDX1* or *NKX6.1* in pancreatic progenitors (39) and even to induce transdifferentiation of human pancreatic non-endocrine tissue (e.g., ductal-mediated beta cell regeneration) to glucose-responsive beta like cells (18, 27). Components of the Notch pathway were also enriched after miR-30d-5p mimic treatment (*NOTCH3* up, *DDX5/DDX17* down), suggesting a potential association with progenitor activation (40, 41). Finally, the miR-30d-5p mimic was associated with increased expression of *IL22RA1*, which promotes beta cell regeneration and mitigates inflammation and ER/oxidative stress (42), reduced expression of the pro-inflammatory transcription factor STAT5B, greater MAPK12 expression and stress responsiveness, and enriches the antioxidant regulator FOXO3, which helps protect pancreatic beta cells from oxidative damage (3, 43).

Our *in vivo* experiments in the NOD mice further support a protective role for miR-30d-5p in autoimmune diabetes. Treatment with the mimic delayed disease onset consistent with prior evidence linking low miR-30d-5p levels to increased islet infiltration and heightened autoimmunity (13). The incidence analysis was concluded at 23 weeks, a time point that captures the main window of disease onset in the NOD model, where incidence typically plateaus between 20–25 weeks under our colony conditions. The delayed disease onset may reflect, at least in part, beta cell recovery and immunomodulatory effects, similar to those seen in humans treated with the first approved immunomodulatory therapy for type 1 diabetes, Teplizumab (44). Consistent with this, the treatment was associated with changes in the frequency of peripheral leukocyte subsets, including an increased percentage of CD4 and CD8 T lymphocytes characterized by elevated PD-1 expression, and a reduced frequency of NK cells. The observed increase in PD-1 expression likely reflects a state of T cell exhaustion, which is known to limit autoimmune responses. This finding aligns with our previous observation that *in vivo* inhibition of miR-30d-5p leads to reduced PD-1 expression on splenocytes (13), further supporting an association between this miRNA and the modulation of immune activation and delayed onset of autoimmune diabetes. Interestingly, we previously reported that NK cells play a critical role in the pancreas for the acceleration of type 1 diabetes in the NOD model (45). These results suggest that miR-30d-5p–mediated reduction of NK cells may contribute to delayed autoimmune diabetes onset by dampening inflammation and reducing early innate activation. Importantly, miR-30d-5p has also been reported to be modulated in metabolic tissues, including pancreatic islets, in the context of high-fat diet–induced metabolic stress, obesity, and type 2 diabetes (46). However, our study was performed in the autoimmune NOD model, which is not associated with metabolic overload or obesity, and therefore direct extrapolation between metabolic and autoimmune contexts should be made with caution. Within this framework, increased miR-30d-5p may enhance PD-1–mediated T cell-inhibition, limiting effector functions and insulitis, thereby contributing to a slower autoimmune progression and reduced beta cell loss.

The inhibition of miR-30d-5p in pre-activated T lymphocytes contributes to the reduction of the expression of immune checkpoint molecules such as CD200, CTLA-4, PD-1, LAG-3, and TIM-3, suggesting a role for miR-30d-5p in promoting inhibitory receptor expression and dampening T-cell responses. CD200 and CTLA-4 emerged as potential targets, but evidence is limited—only CD200 is experimentally validated (TarBase v9.0). In human T lymphocytes, the expression patterns of both genes are inconsistent with them being direct targets, as their expression levels do not increase in the absence of the corresponding miRNA, contrary to what would be anticipated. CTLA-4 and PD-1 are the most well-studied immune checkpoint receptors and are known to downregulate T-cell activation to maintain peripheral tolerance (47). Their interaction with CD80/86 and PD-L1, respectively, prevents T cells from attacking self-tissues. Both pathways are strongly associated with type 1 diabetes pathogenesis (48). For example, blocking PD-1/PD-L1 in NOD mice induces diabetes, while their overexpression reverses it (49). In addition, other immunosuppressive axes also have a key role in type 1 diabetes. The Galectin-9/TIM-3 interaction suppresses Th1 responses and protects against autoimmune diabetes (50), while LAG-3 binding to MHC class II molecules limits T-cell activation and proliferation. LAG-3^-/-^ NOD mice develop diabetes at an accelerated rate with 100% incidence, underscoring its role in restraining pathogenic CD4^+^ T cells (51). Polymorphisms in the CTLA-4 gene have also been associated with an increased susceptibility to developing type 1 diabetes, especially in the pediatric population (52). No changes in CD25 or FoxP3 were observed in this study, whereas our previous *in vivo* work showed an increase in CD4^+^CD25^+^FoxP3^+^ T cells at 10 days after treatment (13), suggesting a delayed effect of miR-30d-5p on Tregs. Therefore, miR-30d-5p modulation was associated with the expression of different inhibitory receptors on T cells, and the upregulation of this miRNA during the PR phase may contribute to the immunoregulatory processes taking place during this period, as previously described (53). Supporting this, an inverse relationship between PD-1 expression and glucose uptake in CD8^+^ T cells was observed during PR in children (54), highlighting how inhibitory pathways impair glycolysis, a key metabolic pathway for T-cell activation (55). Moreover, the triggering of inhibitory receptors like PD-1, CTLA-4, LAG-3, TIM-3 and TIGIT on T lymphocytes results in a progressive loss of effector function, TCR desensitization, and failure to develop memory (56), a pattern reminiscent of the “exhausted” phenotype described when co-stimulatory signals are lacking (57). Thus, the possible association of miR-30d-5p with an exhausted phenotype may elucidate a protective mechanism. Notably, miR-30d-5p deficiency increases CD45RA^+^ subsets, linked to naïve or TEMRA phenotypes. Elevated miR-30d-5p during the PR phase may limit TEMRA differentiation in CD8^+^ T cells, which are both cytotoxic and proinflammatory (58). Finally, regarding T cell function, miR-30d-5p inhibition reduced IFN-γ levels, a cytokine that triggers PD-L1 upregulation in beta cells, limiting autoreactive T-cell activity (59). In addition, genetic knockout of IFN-γ in CAR T cells reduces the expression of immune checkpoint molecules such as CTLA-4, PD-1, LAG-3 and TIM-3 (60, 61). Given that IFN-γ also acts in an autocrine manner, reduced IFN-γ secretion and signaling on T cells may be associated with decreased expression of immune checkpoint molecules.

This study has several limitations. First, we focused on miR-30d-5p in type 1 diabetes without assessing potential effects in other systems, which may be relevant given miRNA pleiotropy. Second, while HPSs are a valuable model, donor heterogeneity and uneven islet distribution complicate data interpretation. In fact, experiments using whole pancreas tissue include only 1-2% of endocrine cells, which is a further limitation. Therefore, functional experiments were also performed to specifically assess the function of insulin-producing beta cells. Third, in NOD mice, the vehicle group showed lower-than-expected diabetes incidence, a phenomenon linked to seasonal variation (62). Finally, transfection efficiency assessed by flow cytometry using fluorescently labeled miRNA may be underestimated due to limited sensitivity, which could contribute to the discrepancy observed with miR-30d-5p expression levels measured by RT-qPCR. In addition, non-specific effects of miRNA mimics and inhibitors should also be considered, as even unrelated controls can alter gene expression (63). Although RNA-seq analysis was performed, pathway enrichment (including KEGG) did not yield robust or statistically significant pathway associations, limiting mechanistic interpretation at the systems level. Importantly, while our data support associations between miR-30d-5p modulation and immune and beta cell–related outcomes, they do not demonstrate direct causality.

Despite these limitations, our integrative approach combining human samples and *in vivo* models provides novel insights into the therapeutic potential of miR-30d-5p in type 1 diabetes. Although no miRNA-based therapies have been approved by the FDA to date, multiple clinical trials are actively investigating their clinical applicability (64). A strength of this study is the use of human samples, including pancreatic tissue from non-diabetic donors and lymphocytes from type 1 diabetes patients. Still, peripheral T cell analysis may not fully recapitulate the properties of islet-infiltrating T cells, as only a minority of circulating T cells are specific for beta cell antigens and traffic to the pancreatic islets. Future studies examining the role of miR-30d-5p in antigen-presenting cells, such as dendritic cells and macrophages, may help to further define its broader immunoregulatory functions in type 1 diabetes. Overall, integrating peripheral immune cells and human pancreatic tissue data enhances the translational relevance of our study and provides a more comprehensive view of miR-30d-5p-associated effects.

In summary, we identified a potential role for miR-30d-5p in association with immune modulation and beta cell homeostasis. Importantly, we demonstrated the feasibility of HPSs transfection with miRNA mimics and inhibitors as an experimental strategy to study human islet biology. The findings presented here provide insight into immune–epigenetic interactions during the PR phase of type 1 diabetes and suggest potential therapeutic avenues for beta cell preservation and regeneration, contributing to a better understanding of this disease stage.

## Data Availability

The datasets generated and/or analyzed during the current study are available in the GEO repository, under accession number GSE316801 and/or are accessible by the corresponding author upon reasonable request.

## Supplementary material

Supplementary Figure 1 miR-30d-5p inhibitor’s delivery to human pancreatic slices: target suppression and assessment of cell viability over time. **(A)** Time-course fold change in the miR-30d-5p expression using basal transcription (complete medium-cultured HPSs) as the standard value. Expression of miR-30d-5p was analyzed by single-assay RT-qPCR 24, 48, 72 and 168 hours after transfection with 200, 300 or 400 nM of miRNA inhibitor or 100 nM of the control inhibitor (negative control, NC). Blue bars represent slices retransfected at 96 hours with another 100 nM of miRNA inhibitor or NC before analyzing miR-30d-5p expression at 168 hours. The miRNA expression signal was normalized to U6 expression. Values are expressed as 2^-ΔΔCt^. N = 1 pancreas, hence no error bars. **(B)** Representative staining showing tissue viability 24 and 168 hours after transfection with 200 or 300 nM of miR-30d-5p inhibitor. Images showing mitochondrial staining (purple) and miRNA inhibitor coupled with FAM (green). Scale: 50 μm for all panels. **(C)** Representative immunostaining showing tissue viability at the time of tissue slicing (basal), and at days 6 and 10 of culture. Images showing live (green) and dead (red) regions. **(D)** Representative immunofluorescence images of human pancreatic slices (day 6) co-transduced with HIP-Cre and loxP-dsRED-loxP-BFP-2A-GCaMP6, and transfected with miR-30d-5p inhibitor, mimic, or controls. BMP-7 (positive control) was added for 5 days and then withdrawn; negative control (NC) slices were only transduced. Blue (BFP) marks β-cells, red (dsRED) non-β-cells, and violet (merge) indicates newly formed β-cells. Scale: 50 μm for all panels.

Supplementary Figure 2 Transcriptomic changes after miR-30d-5p modulation. **(A)** PCA of gene expression after correcting for the batch effect. Principal component 1 and principal component 2 are represented on the x-axis and y-axis, respectively. **(B–D)** Volcano plots of gene expression in the I vs C, M vs C and I vs M comparisons. The control group comprises both CI and CM-treated slices. Lines indicate log(FC) (x−axis) and -log10(FDR) (y−axis) cut-offs. Blue and red dots indicate significantly downregulated and upregulated genes, respectively, and grey dots indicate non-significantly different expression levels. Log(FC) >0.5 for upregulated DEGs, and log(FC) <0.5 for downregulated DEGs. FDR adjusted p-value ≤0.05.

Supplementary Figure 3 Representative gating strategy for mouse T lymphocyte and leukocyte panels. **(A)** Representative plots corresponding to the mouse T cells panel. Lymphoid cells were selected using Forward Scatter (FSC) and Side Scatter (SSC), followed by a singlet exclusion using FSC-Area and FSC-high. After CD3^+^ cell gating (CD3 and FSC-A), cells were split between CD4^+^ and CD8^+^ T lymphocytes. T regulatory cells were selected as FOXP3^+^ cells from CD4^+^ T lymphocytes. Maturation of CD4^+^ and CD8^+^ lymphocytes was determined using CD62L and CD44 markers and was split into 3 subsets: effector (CD44^+^CD62L^-^); central memory (CD44^+^ CD62L^+^); and naïve (CD44^-^ CD62L^+^). PD-1^+^ cells were assessed from CD4^+^, CD8^+^ and regulatory T lymphocytes. **(B)** Representative plots corresponding to the mouse Leukocyte panel. Lymphoid cells were selected using Forward Scatter (FSC) and Side Scatter (SSC), followed by singlet exclusion using FSC-Area and FSC-high. Viable cells were selected as negative 7-aad cells. B lymphocytes were selected as CD19^+^ cells, T lymphocytes were selected as CD3^+^ cells and other immune cells were gated as CD3^-^ CD19^-^ cells. NKT cells were assessed inside T lymphocytes as nkp44^+^ cells. From the double negative gate, we determined the percentage of dendritic cells as CD11c^hi^ MHC-II^hi^, and NK cells as nkp44^+^ MHC-II^-^ cells. NK cells were split into 2 subsets: effector (CD11b^+^ CD27^-^) and regulatory (CD11b^-^ CD27^+^).

Supplementary Figure 4 miR-30d-5p inhibition decreases the percentage of CD4^+^ and CD8^+^ T lymphocytes expressing inhibitory molecules. (A) Relative expression of miR-30d-5p on human T cells (sorted CD4^+^, red; sorted CD8^+^ blue; sorted Treg, lilac) from remitter (PR, n=10) and non-remitter (non-PR, n=6) patients with type 1 diabetes (T1D). miRNA expression signal was normalized to 5S rRNA. Values are expressed as 2^-ΔCt^. Data are presented as mean ± SD. *p <0.05, **p <0.01, ****p <0.0001, Mann-Whitney test. (B) Absolute cell counts 4 and 24 hours after electroporation with different concentrations of miR-30d-5p inhibitor coupled with FAM (n=3–6). Data are presented as mean ± SD. (C-F) Percentage of CD4^+^ (up) and CD8^+^ (down) T lymphocytes expressing CD226, CD200, CTLA-4, and TIM-3 obtained from T1D (n=7) and control (n=6) subjects. (G-I) Percentage of CD4^+^ (up) and CD8^+^ (down) T lymphocytes expressing PD-1, TIGIT, and LAG-3 obtained from T1D (n=7) and control (n=6) subjects. From C to I, within each group, grey dots represent T lymphocytes simply electroporated (mock), light blue dots represent T cells electroporated with a control inhibitor (CI) and dark blue dots with a miR-30d-5p inhibitor. ns ≥0.05, *p <0.05, **p <0.01, ***p <0.001 repeated measures two-way ANOVA with the Geisser-Greenhouse correction and Tukey’s multiple comparisons test.

Supplementary Figure 5 Representative gating strategy for human T lymphocyte activation, maturation and checkpoint stages. **(A)** Representative plots corresponding to the activation and maturation status of human T cells. Lymphoid cells were selected using Forward Scatter (FSC) and Side Scatter (SSC), followed by a singlet exclusion using FSC-Area and FSC-high. Viable cells were selected as negative FV575 cells and CD3^+^ cells were split into CD8^+^ and CD4^+^ T lymphocytes. T regulatory cells (Tregs) were selected as CD25^+^FOXP3^+^ from CD4^+^ T lymphocytes. Maturation of CD4 and CD8 T lymphocytes was assessed using CCR7 and CD45RA to split into four different subsets: Naïve T cells (CD45RA^+^CCR7^+^), Central Memory T cells (CD45RA^-^CCR7^+^), Effector Memory T cells (CD45RA^-^CCR7^-^) and Terminally Differentiated Effector Memory T cells (TEMRA, CD45RA^+^CCR7^-^). Activation of CD4^+^ T cells, CD8^+^ T cells and Tregs was gated as CD69^+^ and/or CD25^+^ cells from each parent. **(B)** Representative plots corresponding to the analysis of inhibitory and activation molecules on T lymphocytes. Lymphoid cells were selected using FSC and SSC, followed by a singlet exclusion using FSC-Area and FSC-high. Viable cells were selected as negative FV575 cells and CD3^+^ cells were split into CD8^+^ and CD4^+^ T lymphocytes. Percentages and Median Fluorescence Intensity (MFI) of LAG-3, CD200, CD226, PD-1, TIGIT, TIM-3 and CTLA-4 were assessed for each subset.

Supplementary Figure 6 Representative plots corresponding to the Flow cytometry analysis of CD200 expression in T cell subsets from T1D patients and healthy donors. Representative flow cytometry dot plots and corresponding histograms showing the frequency and expression levels of CD200 in CD4+ (top panels) and CD8+ (bottom panels) T cells. Peripheral blood mononuclear cells (PBMCs) from Healthy Donors and T1D patients were evaluated under three experimental conditions: Mock, CI (Control inhibitor), and Inhibitor. Gating strategy identifies the positive population for CD200 (boxes in dot plots), with arrows indicating the fluorescence intensity distribution in the histograms (Count vs. Comp-G575-A). Numbers within the dot plots represent the percentage of positive cells for the gated population.

Supplementary Figure 7 Effect of miR-30d-5p inhibition on T lymphocyte phenotype and proliferation. **(A)** Percentage of CD4^+^ (left and middle) and CD8^+^ (right) T lymphocytes expressing the markers CD45RA and CCR7. **(B)** Representative plots corresponding to the maturation status of T cells in patients with T1D and control subjects. Axis are CCR7 and CD45RA. **(C)** Percentage of CD4^+^ and CD8^+^ T lymphocytes expressing the activating marker CD69. **(D)** Percentage of total (CD4^+^CD25^+^FoxP3^+^) Tregs and activated (CD69^+^) Tregs. From A to C, within each group, grey dots represent T lymphocytes simply electroporated (mock), light blue dots represent T cells electroporated with a control inhibitor (CI) and dark blue dots with a miR-30d-5p inhibitor. T lymphocytes were obtained from type 1 diabetes (T1D, n=7) and control (n=6) subjects. **(E)** Percentage of CD3^+^, CD4^+^ and CD8^+^ T lymphocyte proliferation. T lymphocytes from controls (n=6) or T1D patients (n=5) previously activated and electroporated with no oligonucleotides (mock, blue bars), control inhibitor (CI, yellow bars) or miR-30d-5p inhibitor (orange bars) were stained with CellTrace Violet (CTV) and only re-cultured (light colors) or re-stimulated with either IL-2 (medium colors) or antibodies against CD3/CD28/CD2 plus IL-2 (dark colors) for 2 days. T cell proliferation was measured as the percentage of CTV^low^ cells. From A to D, ns ≥0.05 and *p <0.05, repeated measures two-way ANOVA with the Geisser-Greenhouse correction and Tukey’s multiple comparisons test.

Supplementary Table 1 Antibodies used for the staining of mouse splenocytes. * Intracellular antibody.

Supplementary Table 2 Clinical and metabolic data of remitter and non-remitter patients with type 1 diabetes. Data presented as mean ± SD. Mann-Whitney test to compare control subjects and patients with type 1 diabetes. BMI, Body Mass Index; Ctrl, controls; HbA1c, glycated hemoglobin; IDAA1c, insulin dose-adjusted HbA1c; PR, partial remission; T1D dx, type 1 diabetes diagnosis.

Supplementary Table 3 List of antibodies used for T cell immunophenotyping.* Intracellular antibody.

Supplementary Table 4 Clinical and metabolic data of patients with type 1 diabetes and controls. Data presented as mean ± SD. Mann-Whitney test to compare control subjects and patients with type 1 diabetes. BMI, Body Mass Index; Ctrl, controls; HbA1c, glycated hemoglobin; IDAA1c, insulin dose-adjusted HbA1c; ND, not determined; T1D dx, type 1 diabetes diagnosis.
