# Supplementary material for: miR-30d-5p promotes beta cell recovery and immunomodulation in type 1 diabetes
Source: Front Endocrinol (Lausanne). 2026 Jun 2;17:1838666. doi: 10.3389/fendo.2026.1838666 (PMC13311865; doi:10.3389/fendo.2026.1838666)
Supplement: Supplementary Figure 1 — miR-30d-5p inhibitor’s delivery to human pancreatic slices: target suppression and assessment of cell viability over time. (A) Time-course fold change in the miR-30d-5p expression using basal transcription (complete medium-cultured HPSs) as the standard value. Expression of miR-30d-5p was analyzed by single-assay RT-qPCR 24, 48, 72 and 168 hours after transfection with 200, 300 or 400 nM of miRNA inhibitor or 100 nM of the control inhibitor (negative control, NC). Blue bars represent slices retransfected at 96 hours with another 100 nM of miRNA inhibitor or NC before analyzing miR-30d-5p expression at 168 hours. The miRNA expression signal was normalized to U6 expression. Values are expressed as 2-ΔΔCt. N = 1 pancreas, hence no error bars. (B) Representative staining showing tissue viability 24 and 168 hours after transfection with 200 or 300 nM of miR-30d-5p inhibitor. Images showing mitochondrial staining (purple) and miRNA inhibitor coupled with FAM (green). Scale: 50 μm for all panels. (C) Representative immunostaining showing tissue viability at the time of tissue slicing (basal), and at days 6 and 10 of culture. Images showing live (green) and dead (red) regions. (D) Representative immunofluorescence images of human pancreatic slices (day 6) co-transduced with HIP-Cre and loxP-dsRED-loxP-BFP-2A-GCaMP6, and transfected with miR-30d-5p inhibitor, mimic, or controls. BMP-7 (positive control) was added for 5 days and then withdrawn; negative control (NC) slices were only transduced. Blue (BFP) marks β-cells, red (dsRED) non-β-cells, and violet (merge) indicates newly formed β-cells. Scale: 50 μm for all panels. [file DataSheet1.pdf]

SUPPLEMENTARY MATERIAL

miR-30d-5p promotes beta cell recovery and immunomodulation in type 1 diabetes

Laia Gomez-Muñoz[1], David Perna-Barrull[1], Dagmar Klein[2], Silvia Alvarez-Cubela[2], Gerard Godoy-Tena[3,4], Daniel A Cook[1], Catalina Quimper Voto-Bernales[2], Mayur Doke[2], Marta Murillo[5], Aina Valls[5], Ricardo Luis Pastori[2], Juan Dominguez-Bendala[2], Marta Vives-Pi[*1]

[1]Immunology Department, Germans Trias i Pujol Research Institute (IGTP) and University Hospital (HGTiP), Autonomous University of Barcelona, 08916 Badalona, Spain.
[2]Diabetes Research Institute, University of Miami Miller School of Medicine, Miami, FL 33136, USA.
[3]Laboratory of Tumor Inflammation and Angiogenesis, Center for Cancer Biology, VIB, Leuven, Belgium.
[4]Laboratory of Tumor Inflammation and Angiogenesis, Center for Cancer Biology, Department of Oncology, KU Leuven, Leuven, Belgium.
[5]Pediatrics Department, Germans Trias i Pujol Research Institute (IGTP) and University Hospital (HGTiP), Autonomous University of Barcelona, 08916 Badalona, Spain.

Corresponding author: Marta Vives-Pi, Immunology Department, Germans Trias i Pujol Research Institute. Carretera Canyet s/n., 08916 Badalona, Spain. Phone: +34 935 543 050; E-mail address: mvives@igtp.cat

SUPPLEMENTARY FIGURES 1-7

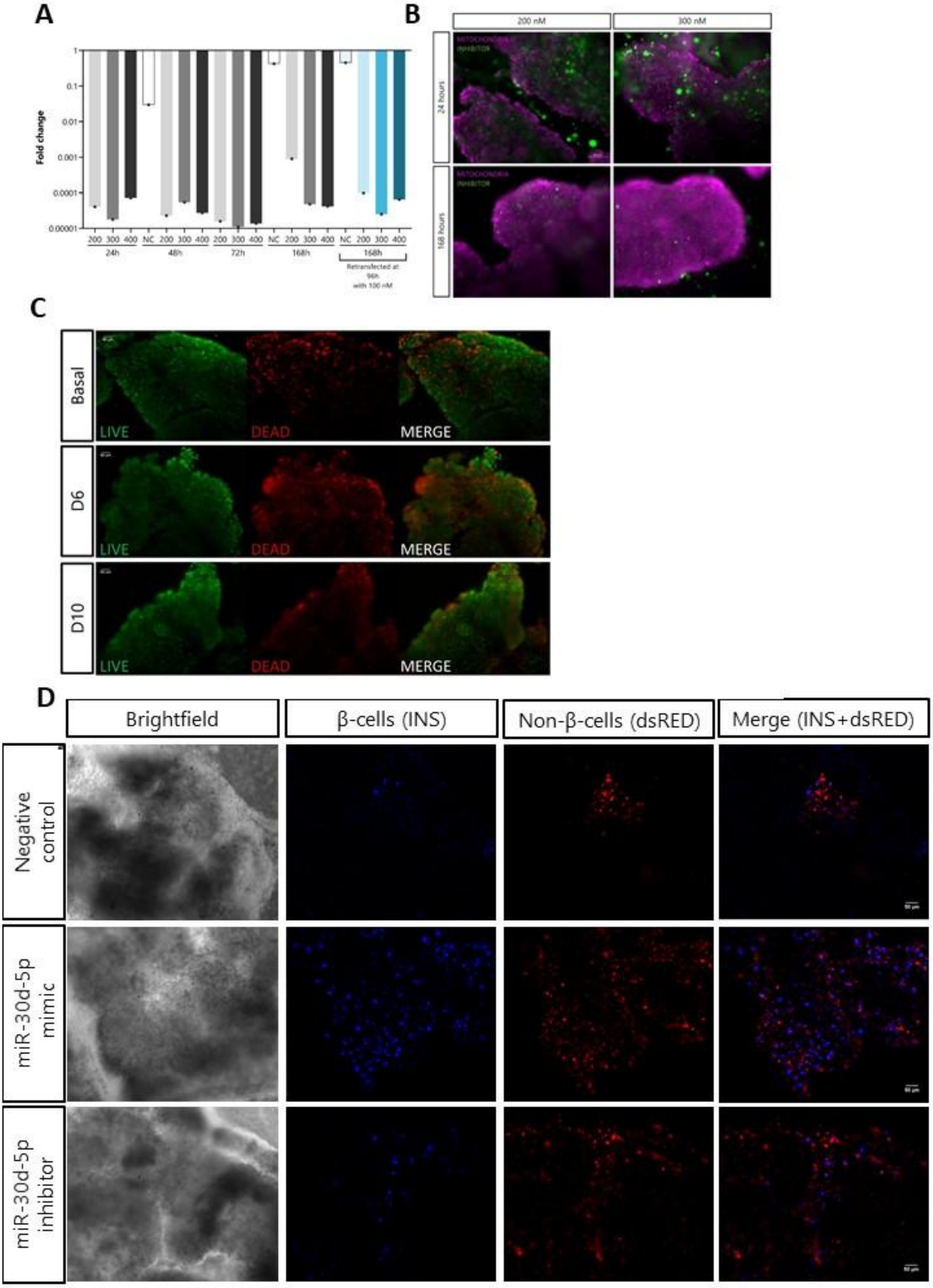

Supplementary Figure 1. miR-30d-5p inhibitor's delivery to human pancreatic slices: target suppression and assessment of cell viability over time. (A) Time-course fold change in the miR-30d-5p expression using basal transcription (complete medium-cultured HPSs) as the standard value. Expression of miR-30d-5p was analyzed by single-assay RT-qPCR 24, 48, 72 and 168 hours after transfection with 200, 300 or 400 nM of miRNA inhibitor or 100 nM of the control inhibitor (negative control, NC). Blue bars represent slices retransfected at 96 hours with another 100 nM of miRNA inhibitor or NC before analyzing miR-30d-5p expression at 168 hours. The miRNA expression signal was normalized to U6 expression. Values are expressed as $2^{-\Delta\Delta Ct}$. N=1 pancreas, hence no error bars**. (B)** Representative staining showing tissue viability 24 and 168 hours after transfection with 200 or 300 nM of miR-30d-5p inhibitor. Images showing mitochondrial staining (purple) and miRNA inhibitor coupled with FAM (green). Scale: 50 µm for all panels. **(C)** Representative immunostaining showing tissue viability at the time of tissue slicing (basal), and at days 6 and 10 of culture. Images showing live (green) and dead (red) regions. **(D)** Representative immunofluorescence images of human pancreatic slices (day 6) co-transduced with HIP-Cre and loxP-dsRED-loxP-BFP-2A-GCaMP6, and transfected with miR-30d-5p inhibitor, mimic, or controls. BMP-7 (positive control) was added for 5 days and then withdrawn; negative control (NC) slices were only transduced. Blue (BFP) marks β-cells, red (dsRED) non-β-cells, and violet (merge) indicates newly formed β-cells. Scale: 50 µm for all panels.

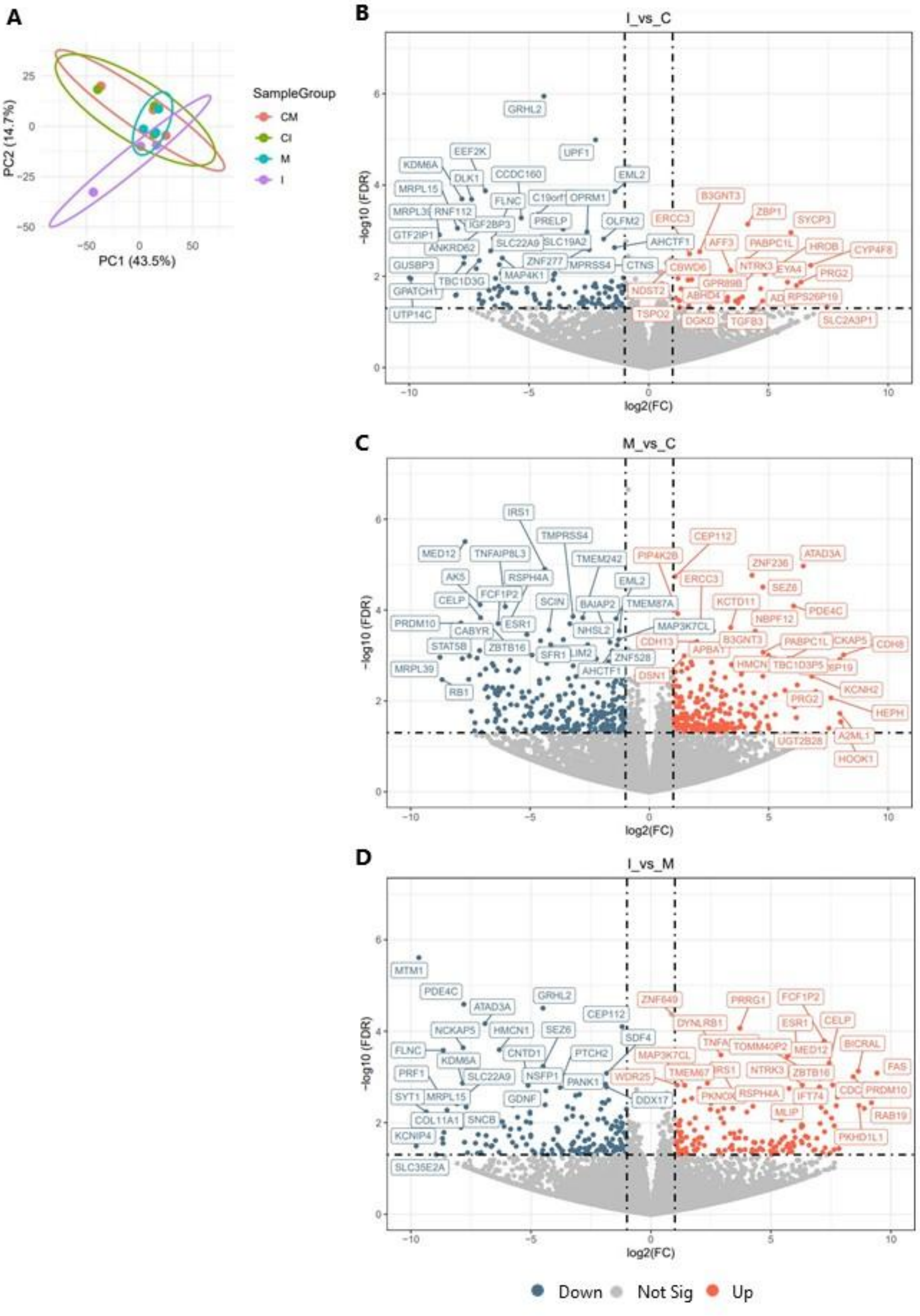

Supplementary Figure 2. Transcriptomic changes after miR-30d-5p modulation. (A) PCA of gene expression after correcting for the batch effect. Principal component 1 and principal component 2 are represented on the x-axis and y-axis, respectively. **(B-D)** Volcano plots of gene expression in the I vs C, M vs C and I vs M comparisons. The control group comprises both CI and CM-treated slices. Lines indicate log(FC) (x–axis) and -log10(FDR) (y–axis) cut-offs. Blue and red dots indicate significantly downregulated and upregulated genes, respectively, and grey dots indicate non-significantly different expression levels. Log(FC) >0.5 for upregulated DEGs, and log(FC) <0.5 for downregulated DEGs. FDR adjusted p-value ≤0.05.

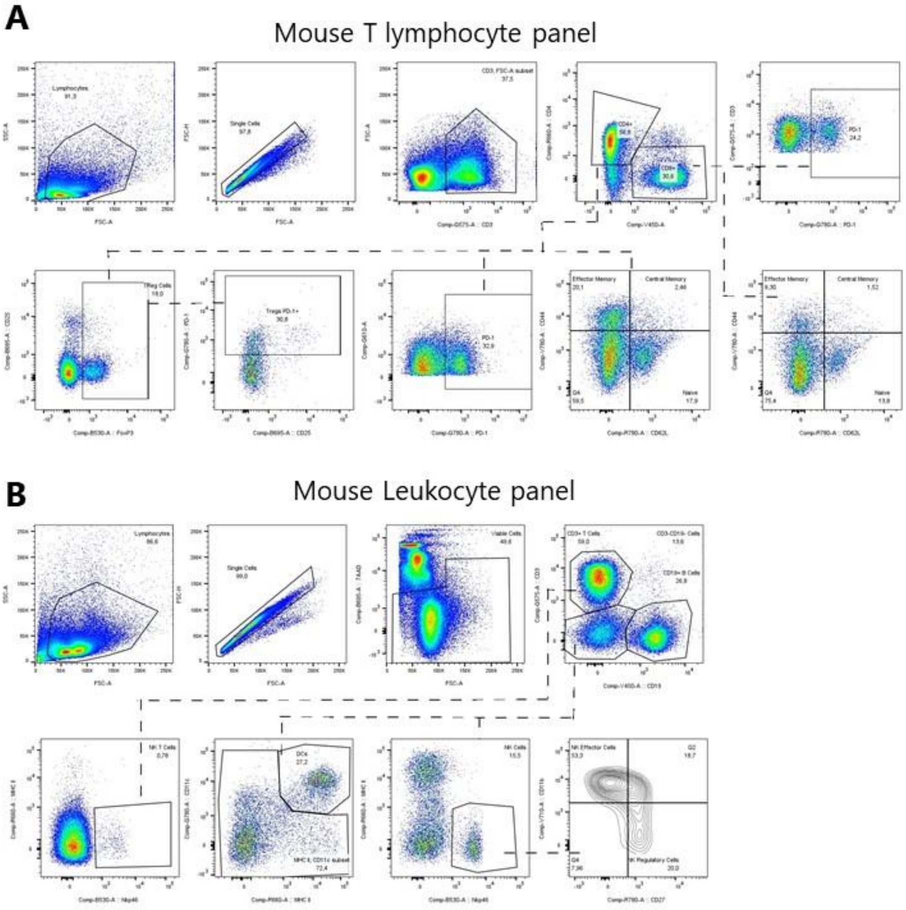

Supplementary Figure 3. Representative gating strategy for mouse T lymphocyte and leukocyte panels. (A) Representative plots corresponding to the mouse T cells panel. Lymphoid cells were selected using Forward Scatter (FSC) and Side Scatter (SSC), followed by a singlet exclusion using FSC-Area and FSC-high. After $CD3^{+}$ cell gating (CD3 and FSC-A), cells were split between $CD4^{+}$ and $CD8^{+}$ T lymphocytes. T regulatory cells were selected as $FOXP3^{+}$ cells from $CD4^{+}$ T lymphocytes. Maturation of $CD4^{+}$ and $CD8^{+}$ lymphocytes was determined using CD62L and CD44 markers and was split into 3 subsets: effector ($CD44^{+}CD62L^{-}$); central memory ($CD44^{+}$ $CD62L^{+}$); and naïve ($CD44^{-}$ $CD62L^{+}$). $PD\text{-}1^{+}$ cells were assessed from $CD4^{+}$, $CD8^{+}$ and regulatory T lymphocytes. **(B)** Representative plots corresponding to the mouse Leukocyte panel. Lymphoid cells were selected using Forward Scatter (FSC) and Side Scatter (SSC), followed by singlet exclusion using FSC-Area and FSC-high. Viable cells were selected as negative 7-AAD cells. B lymphocytes were selected as $CD19^{+}$ cells, T lymphocytes were selected as $CD3^{+}$ cells and other immune cells were gated as $CD3^{-}$ $CD19^{-}$ cells. NKT cells were assessed inside T lymphocytes as $nkp44^{+}$ cells. From the double negative gate, we determined the percentage of dendritic cells as $CD11c^{hi}$ $MHC\text{-}II^{hi}$, and NK cells as $nkp44^{+}$ $MHC\text{-}II^{-}$ cells. NK cells were split into 2 subsets: effector ($CD11b^{+}$ $CD27^{-}$) and regulatory ($CD11b^{-}$ $CD27^{+}$).

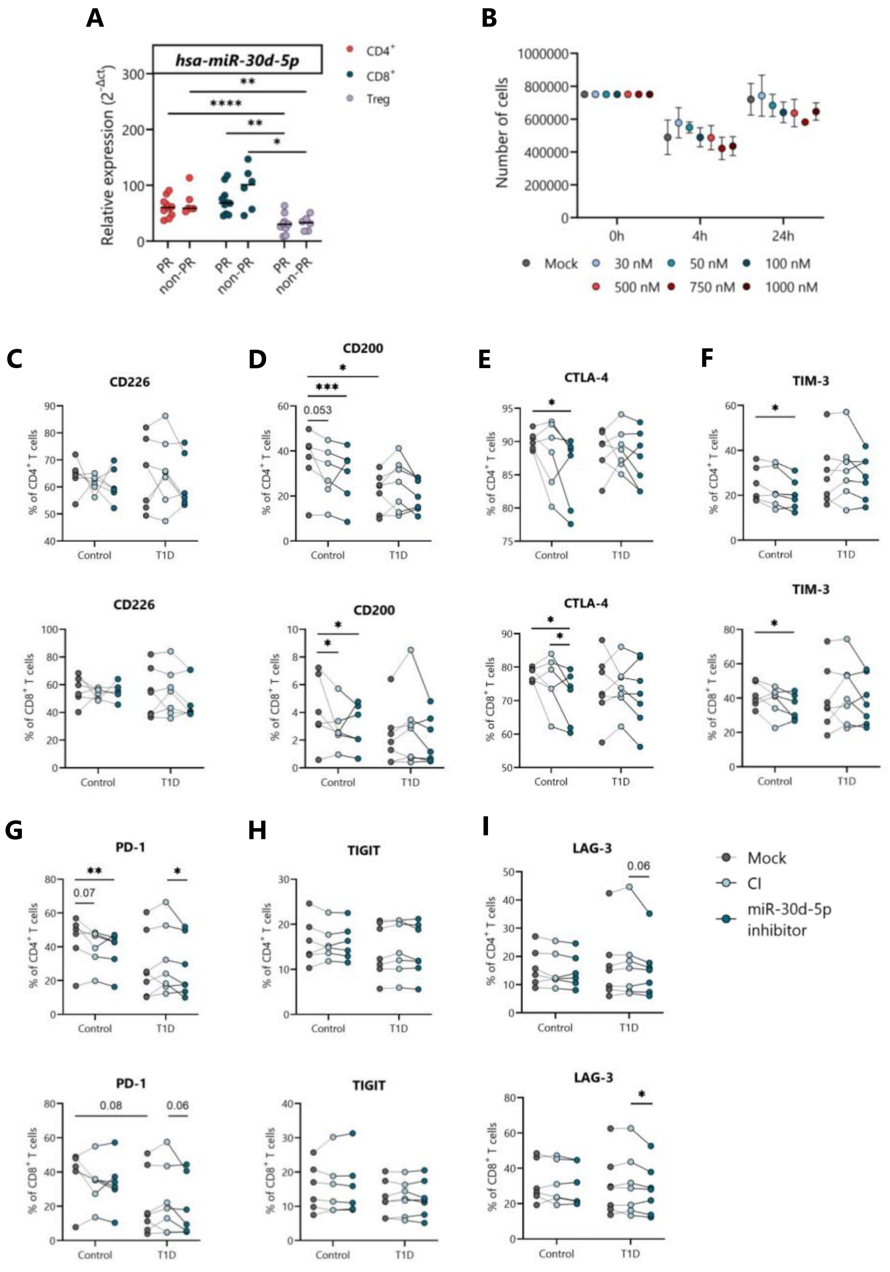

Supplementary Figure 4. miR-30d-5p inhibition decreases the percentage of CD4⁺ and CD8⁺ T lymphocytes expressing inhibitory molecules. (A) Relative expression of miR-30d-5p on human T cells (sorted CD4⁺, red; sorted CD8⁺ blue; sorted Treg, lilac) from remitter (PR, n=10) and non-remitter (non-PR, n=6) patients with type 1 diabetes (T1D). miRNA expression signal was normalized to 5S rRNA. Values are expressed as $2^{-\Delta Ct}$. Data are presented as mean ± SD. *p <0.05, **p <0.01, ****p <0.0001, Mann-Whitney test. **(B)** Absolute cell counts 4 and 24 hours after electroporation with different concentrations of miR-30d-5p inhibitor coupled with FAM (n=3–6). Data are presented as mean ± SD. **(C-F)** Percentage of CD4⁺ (up) and CD8⁺ (down) T lymphocytes expressing CD226, CD200, CTLA-4, and TIM-3 obtained from T1D (n=7) and control (n=6) subjects. **(G-I)** Percentage of CD4⁺ (up) and CD8⁺ (down) T lymphocytes expressing PD-1, TIGIT, and LAG-3 obtained from T1D (n=7) and control (n=6) subjects. From C to I, within each group, grey dots represent T lymphocytes simply electroporated (mock), light blue dots represent T

cells electroporated with a control inhibitor (CI) and dark blue dots with a miR-30d-5p inhibitor. ns ≥0.05, *p <0.05, **p <0.01, ***p <0.001 repeated measures two-way ANOVA with the Geisser-Greenhouse correction and Tukey's multiple comparisons test.

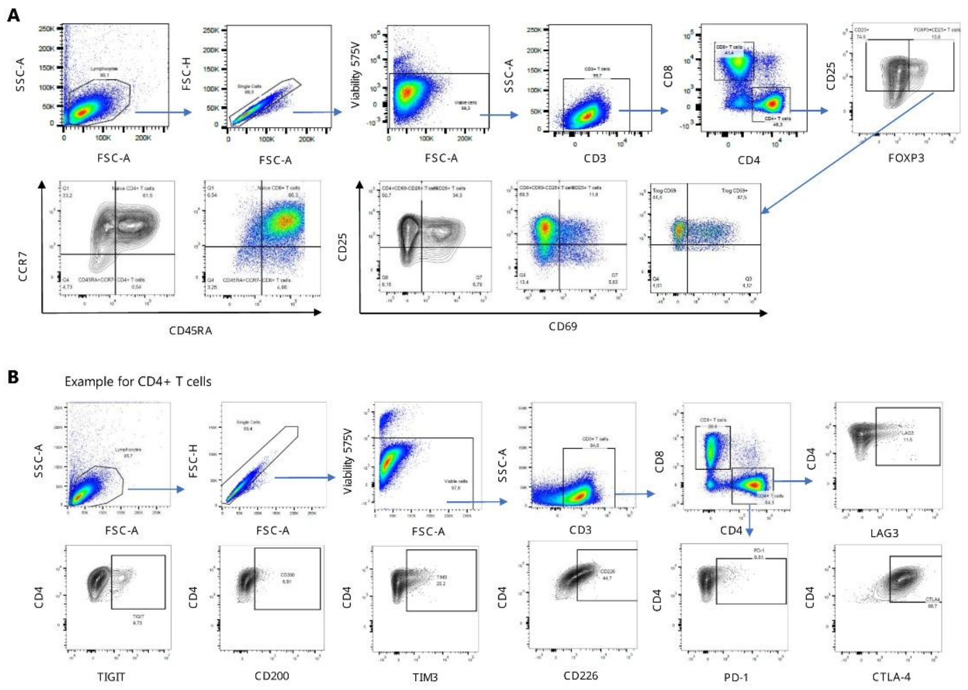

Supplementary Figure 5. Representative gating strategy for human T lymphocyte activation, maturation and checkpoint stages. (A) Representative plots corresponding to the activation and maturation status of human T cells. Lymphoid cells were selected using Forward Scatter (FSC) and Side Scatter (SSC), followed by a singlet exclusion using FSC-Area and FSC-high. Viable cells were selected as negative FV575 cells and $CD3^+$ cells were split into $CD8^+$ and $CD4^+$ T lymphocytes. T regulatory cells (Tregs) were selected as $CD25^+FOXP3^+$ from $CD4^+$ T lymphocytes. Maturation of CD4 and CD8 T lymphocytes was assessed using CCR7 and CD45RA to split into four different subsets: Naïve T cells ($CD45RA^+CCR7^+$), Central Memory T cells ($CD45RA^-CCR7^+$), Effector Memory T cells ($CD45RA^-CCR7^-$) and Terminally Differentiated Effector Memory T cells (TEMRA, $CD45RA^+CCR7^-$). Activation of $CD4^+$ T cells, $CD8^+$ T cells and Tregs was gated as $CD69^+$ and/or $CD25^+$ cells from each parent. **(B)** Representative plots corresponding to the analysis of inhibitory and activation molecules on T lymphocytes. Lymphoid cells were selected using FSC and SSC, followed by a singlet exclusion using FSC-Area and FSC-high. Viable cells were selected as negative FV575 cells and $CD3^+$ cells were split into $CD8^+$ and $CD4^+$ T lymphocytes. Percentages and Median Fluorescence Intensity (MFI) of LAG-3, CD200, CD226, PD-1, TIGIT, TIM-3 and CTLA-4 were assessed for each subset.

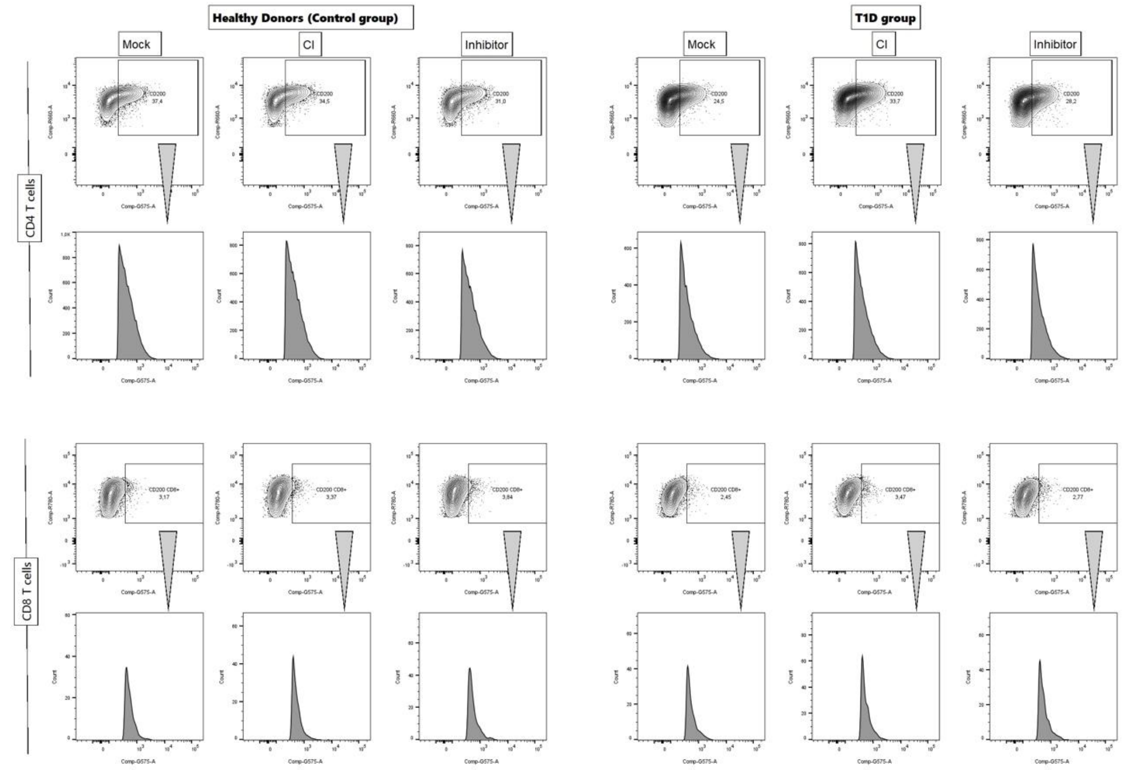

Supplementary Figure 6. Representative plots corresponding to the Flow cytometry analysis of CD200 expression in T cell subsets from T1D patients and healthy donors. Representative flow cytometry dot plots and corresponding histograms showing the frequency and expression levels of CD200 in CD4^{+} (top panels) and CD8^{+} (bottom panels) T cells. Peripheral blood mononuclear cells (PBMCs) from Healthy Donors and T1D patients were evaluated under three experimental conditions: Mock, CI (Control inhibitor), and Inhibitor. Gating strategy identifies the positive population for CD200 (boxes in dot plots), with arrows indicating the fluorescence intensity distribution in the histograms (Count vs. Comp-G575-A). Numbers within the dot plots represent the percentage of positive cells for the gated population.

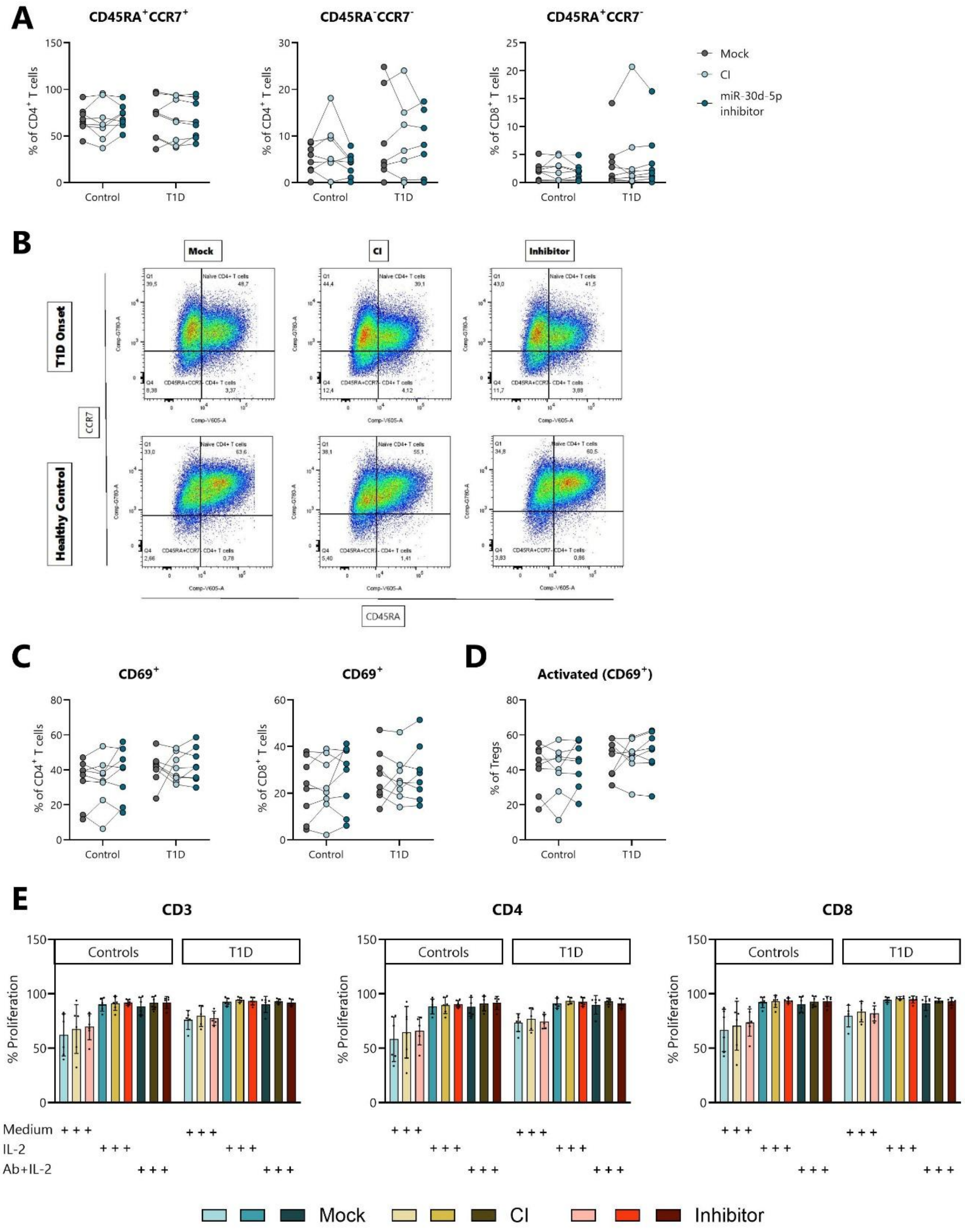

Supplementary Figure 7. Effect of miR-30d-5p inhibition on T lymphocyte phenotype and proliferation. **(A)** Percentage of $CD4^+$ (left and middle) and $CD8^+$ (right) T lymphocytes expressing the markers CD45RA and CCR7. **(B)** Representative plots corresponding to the maturation status of T cells in patients with T1D and control subjects. Axis are CCR7 and CD45RA. **(C)** Percentage of $CD4^+$ and $CD8^+$ T lymphocytes expressing the activating marker CD69. **(D)** Percentage of total ($CD4^+CD25^+FoxP3^+$) Tregs and activated ($CD69^+$) Tregs. From A to C, within each group, grey dots represent T lymphocytes simply electroporated (mock), light blue dots represent T cells electroporated with a control inhibitor (CI) and dark blue dots with a miR-30d-5p inhibitor. T lymphocytes were obtained from type 1 diabetes (T1D, n=7) and control (n=6) subjects. **(E)** Percentage of $CD3^+$, $CD4^+$ and $CD8^+$ T lymphocyte proliferation. T lymphocytes from

controls (n=6) or T1D patients (n=5) previously activated and electroporated with no oligonucleotides (mock, blue bars), control inhibitor (CI, yellow bars) or miR-30d-5p inhibitor (orange bars) were stained with CellTrace Violet (CTV) and only re-cultured (light colors) or re-stimulated with either IL-2 (medium colors) or antibodies against CD3/CD28/CD2 plus IL-2 (dark colors) for 2 days. T cell proliferation was measured as the percentage of CTV^{low} cells. From A to D, ns ≥0.05 and *p <0.05, repeated measures two-way ANOVA with the Geisser-Greenhouse correction and Tukey's multiple comparisons test.

SUPPLEMENTARY TABLES 1-4

Supplementary Table 1. Antibodies used for the staining of mouse splenocytes

	Target	Fluorochrome	Clone	Use	Company
Leukocyte Panel	CD3	PE	500A2	1 µL	BD Bioscience
	CD11b	Brilliant Violet 711	M1/70	0.25 µL	BD Bioscience
	CD11c	PE-Cy7	HL3	1 µL	BD Bioscience
	CD19	Brilliant Violet 450	1D3	1 µL	BD Bioscience
	CD27	APC-Fire 750	LG.3A10	2.5 µL	BioLegend
	NKp46	FITC	29A1.4	0.5 µL	BD Bioscience
	MHC-II	APC	AMS-32.1	1 µL	eBioscience
T Lymphocyte Panel	CD3	PE	500A2	1 µL	BD Bioscience
	CD4	APC	RM4-5	1 µL	BD Bioscience
	CD8	Brilliant Violet 421	53-6.7	0.3 µL	BD Bioscience
	PD-1	PE-Cy7	29F.1A12	2 µL	BioLegend
	CD25	PerCP-Cy5.5	PC61.5	1 µL	eBioscience
	CD44	Brilliant Violet 786	IM7	0.2 µL	BD Bioscience
	CD62L	APC-Cy7	MEL-14	1.6 µL	BioLegend
	FOXP3*	FITC	FJK-16s	1.5 µL	eBioscience

* Intracellular antibody

Supplementary Table 2. Clinical and metabolic data of remitter and non-remitter patients with type 1 diabetes

	**PR**	**No PR**
**N (no. girls)**	10 (1)	6 (2)
**Age at diagnosis (years)**	9.4 ± 4.5	11.2 ± 5.46
**BMI (kg/m^2)**	18.19 ± 3.39	17.71 ± 1.72
**HbA1c (%)**	7.16 ± 0.57	8.32 ± 0.99
**Insulin dose (U/kg/day)**	0.36 ± 0.15	0.63 ± 0.16
**IDAA1c**	8.53 ± 0.65	10.82 ± 0.96
**Basal C-peptide (ng/ml)**	0.59 ± 1.01	0.14 ± 0.35

Data presented as mean ± SD. Mann-Whitney test to compare control subjects and patients with type 1 diabetes. Abbreviations: BMI, Body Mass Index; Ctrl, controls; HbA1c, glycated hemoglobin; IDAA1c, insulin dose-adjusted HbA1c; PR, partial remission; T1D dx, type 1 diabetes diagnosis.

Supplementary Table 3. List of antibodies used for T cell immunophenotyping

	Target	Fluorochrome	Clone	Use/100 µL	Company
**Activation and Maturation Panel (Panel 1)**	CD3	V500	UCHT1	2 µL	BD Biosciences
	CD4	V450	RPA-T4	5 µL	BD Biosciences
	CD8	APC-H7	SK1	5 µL	BD Biosciences
	CD25	PE	M-A251	1 µL	BD Biosciences
	CD69	BV711	FN50	1 µL	BioLegend
	FOXP3*	FITC	206D	2.5 µL	BioLegend
	CCR7	PE-Cy7	3D12	1 µL	BD Biosciences
	CD45RA	BV605	HI100	1 µL	BD Biosciences
**Immune checkpoint T Lymphocyte Panel (Panel 2)**	CD3	V500	UCHT1	2 µL	BD Biosciences
	CD4	APC	EDU-2	5 µL	Immunotools
	CD8	APC-H7	SK1	5 µL	BD Biosciences
	CD200	PE	MRC OX-104	2 µL	BD Biosciences
	TIGIT	BV605	A15153G	1 µL	BioLegend
	TIM-3	PE-Cy7	F38-2E2	5 µL	BioLegend
	LAG-3	PE-Cy5	11C3C65	1 µL	BioLegend
	CTLA-4*	BV421	BNI3	5 µL	BioLegend
	PD-1	BV711	EH12.1	1 µL	BD Biosciences
	CD226	FITC	11A8	1 µL	BioLegend

* Intracellular antibody

Supplementary Table 4. Clinical and metabolic data of patients with type 1 diabetes and controls.

	**Controls**	**T1D dx**
**N (no. girls)**	6 (1)	7 (3)
**Age at diagnosis (years)**	11.5 ± 5.1	10.6 ± 4.5
**BMI (kg/m^2)**	20.9 ± 5.3	17.1 ± 4.5
**HbA1c (%)**	ND	10.9 ± 2.2
**Insulin dose (U/kg/day)**	ND	0.62 ± 0.23
**IDAA1c**	ND	13.7 ± 3.2
**Basal C-peptide (ng/ml)**	ND	0.56 ± 0.42

Data presented as mean ± SD. Mann-Whitney test to compare control subjects and patients with type 1 diabetes. Abbreviations: BMI, Body Mass Index; Ctrl, controls; HbA1c, glycated hemoglobin; IDAA1c, insulin dose-adjusted HbA1c; ND, not determined; T1D dx, type 1 diabetes diagnosis.
